# Synthesis and multifaceted pharmacological activity of novel quinazoline NHE-1 inhibitors

**DOI:** 10.1038/s41598-021-03722-w

**Published:** 2021-12-21

**Authors:** Alexander Spasov, Alexander Ozerov, Pavel Vassiliev, Vadim Kosolapov, Natalia Gurova, Aida Kucheryavenko, Ludmila Naumenko, Denis Babkov, Viktor Sirotenko, Alena Taran, Roman Litvinov, Alexander Borisov, Vladlen Klochkov, Darya Merezhkina, Mikhail Miroshnikov, Georgy Uskov, Nadezhda Ovsyankina

**Affiliations:** 1 Department of Pharmacology & Bioinformatics, Volgograd State Medical University, Volgograd, Russia 400131; 2 Scientific Center for Innovative Drugs, Volgograd State Medical University, Volgograd, Russia 400087; 3 Department of Pharmaceutical & Toxicological Chemistry, Volgograd State Medical University, Volgograd, Russia 400131

**Keywords:** Drug discovery, Medicinal chemistry, Pharmacology

## Abstract

The Na^+^/H^+^ exchanger isoform 1 (NHE-1) attracts ongoing attention as a validated drug target for the management of cardiovascular and ocular diseases owing to cytoprotective, anti-ischemic and anti-inflammatory properties of NHE-1 inhibitors. Herein we report novel NHE-1 inhibitors realized via functionalization of *N*^1^-alkyl quinazoline-2,4(1*H*,3*H*)-dione and quinazoline-4(3*H*)-one with *N*-acylguanidine or 3-acyl(5-amino-1,2,4-triazole) side chain. Lead compounds show activity in a nanomolar range. Their pharmacophoric features were elucidated with neural network modeling. Several compounds combine NHE-1 inhibition with antiplatelet activity. Compound **6b** reduces intraocular pressure in rats and effectively inhibits the formation of glycated proteins. Compounds **3e** and **3i** inhibit pro-inflammatory activation of murine macrophages, LPS-induced interleukin-6 secretion and also exhibit antidepressant activity similar to amiloride. Hence, novel compounds represent an interesting starting point for the development of agents against cardiovascular diseases, thrombotic events, excessive inflammation, long-term diabetic complications and glaucoma.

## Introduction

Na^+^/H^+^ exchangers (NHEs), the solute carrier 9 family (SLC9), are ancient highly conserved transporters that play a pivotal role in regulating intracellular pH by electroneutral exchange of Na^+^ and H^+^ across cellular membranes. The Na^+^/H^+^ exchanger isoform 1 (NHE-1) was the first to be discovered and is the most studied one^1^. NHE-1 is almost ubiquitously expressed and mediates a plethora of cellular processes, thus representing a valuable pharmacological target^2^.

NHE-1 activation in immune cells has a proinflammatory effect^3^. Phagocytosis, cytokine and chemokine release, reactive oxygen species generation depends on intracellular pH^4^. Inhibition of NHE-1 reduced the production of superoxide anion and pro-inflammatory cytokines IL-6, IL-1β and TNF-α induced by LPS in microglia^5^. NHE-1 inhibitor amiloride was shown to ameliorate inflammation and tissue damage in acute lung injury induced with LPS in rats^6^. Additionally, anti-seizure and antidepressant activity of amiloride are also have been linked to NHE-1 inhibition^7^.

Activation of NHE-1 also potentiates the formation of platelet aggregates^8^. Antiplatelet activity of NHE-1 inhibitors is believed to be mediated by inhibition of cytoplasmic Ca^2+^ mobilization and arachidonic acid formation^9^. Previously we have shown that NHE-1 inhibitors reduce the thrombogenic potential of blood in experimental models of cardiovascular pathology^10^.

Na^+^/H^+^ and Cl^-^/HCO_3_^-^ exchangers in epithelial cells of the ciliary body are involved in the first stage of intraocular fluid secretion. Blocking these transporters in the cell culture of bovine pigment and non-pigment epithelium of the ciliary body prevents the absorption of Na^+^ ions and reduces the aqueous humor formation. Hence, NHE-1 inhibitors are considered as a means to reduce intraocular pressure^11^. In addition, NHE-1 prevents intracellular acidosis, plays a role in the proliferation of retinal vascular smooth muscle cells, and may mediate the action of endothelin on vasculature^12,13^.

Additionally, advanced glycation end products (AGEs) induced proliferation of vascular smooth muscle cells is dose-dependently related to the activation of NHE-1^14^. Therefore, antiglycating activity can be considered complementary to NHE-1 inhibition for the prevention and treatment of cardiovascular complications of diabetes.

Compounds incorporating guanidine moiety are known to exhibit diverse biological activity^15^. In particular, guanidine is incorporated in a number of clinically approved and experimental NHE-1 inhibitors, e.g. zoniporide, rimeporide, cariporide and benzoylguanidine^16,17^ or (furan-2-ylcarbonyl)guanidines^18^ derivatives, respectively. Structure of cariporide/NHE-1 complex was recently solved using cryo-EM^19^. It was demonstrated that acylguanidine moiety plays a crucial role in the binding. Guanidine residue forms a cation−π interaction with Phe162 and hydrogen bonds with Asp267 side chain, while carbonyl is also coordinated by the sidechain of Glu346 in the active site of NHE-1.

**Figure 1:**
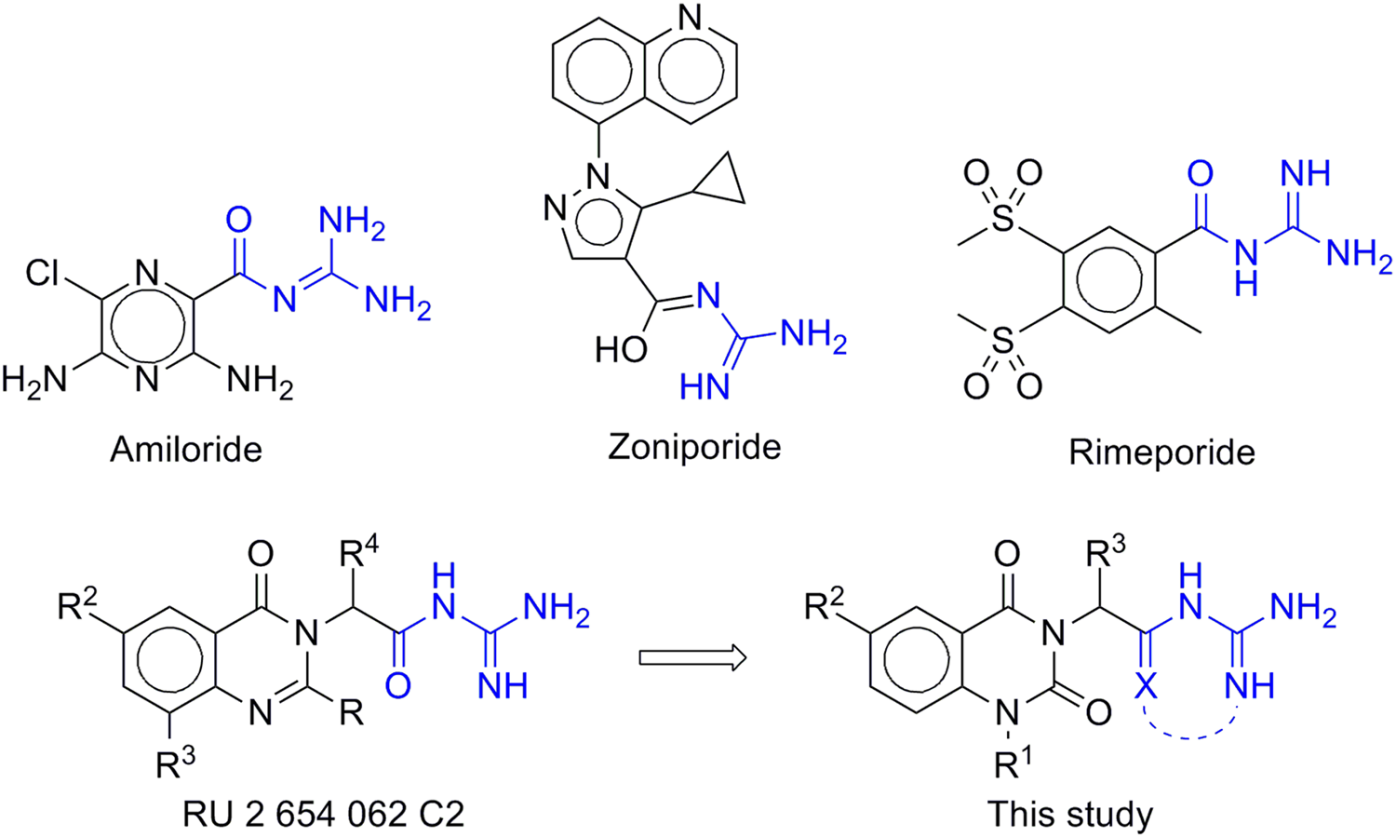
Reference NHE-1 inhibitors and design of the target compounds.

On the other side, quinazoline is a valuable scaffold enriched with biological activity such as anticonvulsant, antimicrobial and antimalarial, antiviral, anti-inflammatory, antituberculosis, enzyme inhibitory, analgesic, and anticancer. Its utility in medicinal chemistry and drug discovery was comprehensevely discussed in several recent reviews^20–23^. Previously, we have reported a series of acylguanidine derivatives of pyrimidine^24^ and quinazoline-4(3*H*)-one^25^ as NHE-1 inhibitors. In this study, we describe the design, synthesis and biological evaluation of their modified analogs, where acylguanidine or its cyclic analog is conjugated with quinazoline-2,4(1*H*,3*H*)-dione or quinazoline-4(3*H*)-one (Fig. 1).

## Results

### Synthesis

**Figure 2:**
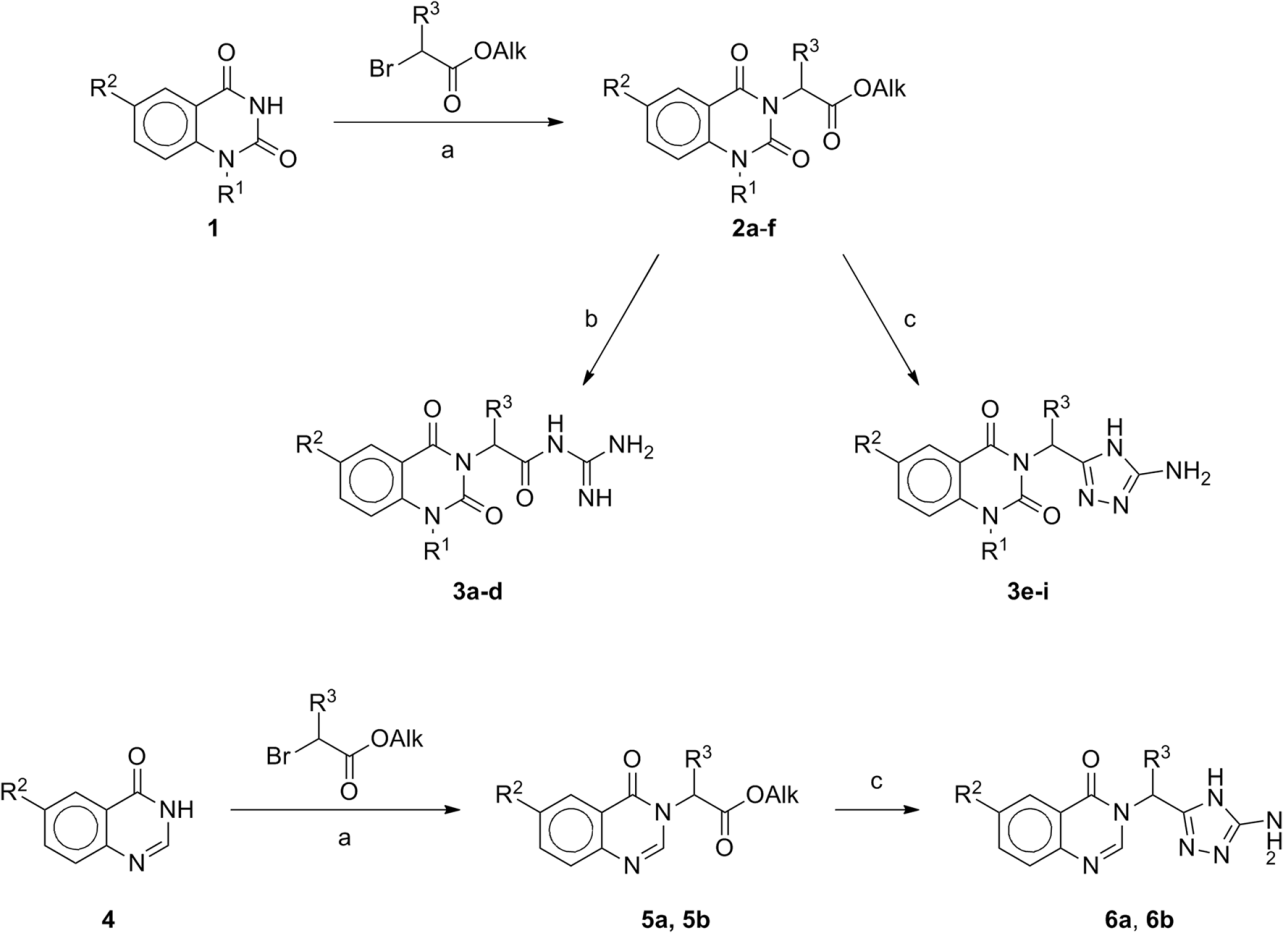
Synthetic route for the target compounds. (**a**) K_2_CO_3_, DMF, rt, 24 h; (**b**) guanidine hydrochloride, EtOH, KOH, reflux, 10 min; (**c**) aminoguanidine carbonate, EtOH, KOH, reflux, 1 h.

Target compounds were realized via the synthetic route shown in Fig. 2. Starting *N*^1^-substituted quinazoline-2,4 (1*H*,3*H*)-diones **1** were readily alkylated with esters of bromoacetic and *D*,*L*-2-bromopropionic acid at room temperature in an anhydrous DMF medium in the presence of the excessive amount of potassium carbonate. Corresponding esters **2a-f** were obtained in 67-81% yield. The next step involved treatment of esters **2a-f** with guanidine generated *in situ* from guanidine hydrochloride and potassium hydroxide in boiling 95% ethanol, which leads to rapid cleavage of the ester bond and formation of *N*-acyl derivatives of guanidine **3a-d** in 57-84% yield.

When aminoguanidine was used as a nucleophilic reagent, which was similarly obtained *in situ* from aminoguanidine carbonate and potassium hydroxide in boiling 95% ethanol, the reaction is accompanied by cyclization to form 5-amino-1,2,4-triazole and leads to quinazoline-2,4(1*H*,3*H*)-dione derivatives **3e-i** with a yield of 60-81%. Ester derivatives of quinazoline-4(3*H*)-one **5a** and **5b** analogously react with aminoguanidine to form 5-amino-1,2,4-triazole derivatives **6a** and **6b**.

### NHE-1 inhibition

Firstly, newly obtained quinazoline-2,4(1*H*,3*H*)-dione and quinazoline-4(3*H*)-one derivatives were assayed for NHE-1 inhibition. Compounds **3a-d** comprising acyclic guanidine fragments showed moderate activity (Table 1). Structure-activity analysis suggests that bulky allyl and benzyl *N*^1^-substituent favors methyl group as R^3^, while small methyl at *N*^1^ can only tolerate unbranched side chain (i.e., R^3^ = H). Bromine at *C*^5^ did not influence the activity. Derivatives **3e-I**, **6a** and **6b** that contain 5-amino-1,2,4-triazole in *N*^3^ side chain generally showed better efficacy (Table 2). Previously made SAR observation is also evident here, since methyl R^3^ in the linker region decreases the NHE-1 inhibitory activity for *N*^1^-methyl-substituted compounds, but improves it for *N*^1^-allyl counterparts. Structural similarity of the most active compounds **3e** and **6a** (R^2^ = R^3^ = H) suggests that R^1^ and *C*^2^ carbonyl are dispensable for NHE-1 inhibition.

**Table 1:** NHE-1 inhibition by novel quinazoline-2,4(1*H*,3*H*)-dione derivatives with acyclic guanidine moiety.


Comp	R^1^	R^2^	R^3^	NHE-1 inhibition at 10 nM, m ± SD, n = 6 (%)	IC_50_ (nM)
**3a**	CH_3_	H	H	33,70 ± 10,23*	292.7
**3b**	CH_3_	H	Me	1,49 ± 4,79^#^	–^a^
**3c**	PhCH_2_	H	H	7,30 ± 5,00^#^	–
**3d**	CH_3_	Br	H	33,21 ± 9,83*	961.4
Rimeporide	–	–	–	34,23 ± 5,91*	–
Amiloride	–	–	–	16,38 ± 2,72*	1230.1
Zoniporide	–	–	–	48,05 ± 7,09*	7.3

Statistical significance: *p < 0,05 vs. negative control; ^#^*p* < 0,05 vs. Zoniporide (1-way ANOVA). ^a^Not tested.

**Table 2:** NHE-1 inhibition by novel quinazoline-2,4(1*H*,3*H*)-dione and quinazoline-4(3*H*)-one derivatives with cyclic guanidine moiety.


Comp	R^1^	R^2^	R^3^	NHE-1 inhibition at 10 nM, m ± SD, n = 6 (%)	IC_50_ (nM)
**3e**	CH_3_	H	H	57,80 ± 3,45*	5.8
**3f**	CH_3_	H	CH_3_	29,76 ± 9,03*	–^a^
**3g**	CH_2_ = CH-CH_2_	H	H	10,39 ± 4,42^#^	–
**3h**	CH_2_ = CH-CH_2_	H	CH_3_	33,24 ± 11,71*	n.d.^b^
**3i**	PhCH_2_	H	H	12,94 ± 2,59*^#^	–
**6a**	-	H	H	54,06 ± 6,38*	6.7
**6b**	-	Br	H	25,81 ± 6,15*	–
Zoniporide	-	-	-	48,05 ± 7,09*	7.3

Statistical significance: *p < 0,05 vs. negative control; ^#^*p* < 0,05 vs. Zoniporide (1-way ANOVA). ^a^Not tested. ^b^Not determined.

### Pharmacophore modeling

**Figure 3:**
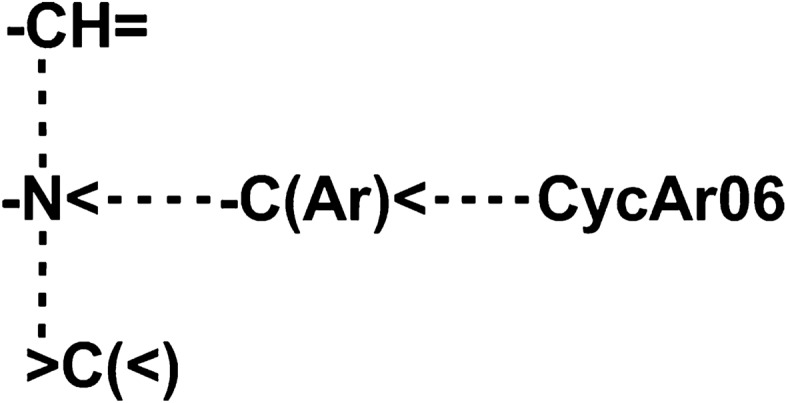
Pharmacophore that defines a high level of NHE-1-inhibitory activity of novel compounds.

To rationalize the obtained SAR and guide further optimization efforts we have undertaken neural network modeling to elucidate the pharmacophoric features of the identified NHE-1 inhibitors. The best performing neural network including 13 most sensitive neurons was obtained after three iterations, during which a total of about 500 neural networks were trained and analyzed (Table 3). For the best neural network model obtained as a result of the third iteration, the correlation coefficient on the combined dataset was R = 0.971 (*p* <5×10^-7^). This model includes four types of QL-descriptors that correspond to the most sensitive neurons and significantly affect the level of NHE-1 inhibitory activity of new compounds: {-N< … -CH=}, Sens = 26.9; {-N< … >C(<)}, Sens = 49.6; {-N< … -C(Ar)<}, Sens = 90.8; {-C(Ar)< … CycAr06}, Sens = 46.3. The combination of these binding points results in a pharmacophore that determines a high level of NHE-1 inhibitory (Fig. 3). Incorporation of the constructed pharmacophore into the structures of the two most active compounds and zoniporide are shown in Table 4. The structures of compound **6a** and zoniporide contain complete pharmacophores of 17 and 24 entries of four types of QL-descriptors of a high level of NHE-1-inhibitory activity, respectively. The structure of compound **3e** includes 27 entries of three types of QL-descriptors of this pharmacophore, and all of the found pharmacophore fragments occur several times.

**Table 3:** Neural networks were obtained after iterative modeling.

Iteration No	Network architecture	Correlation coefficient	
		Train set	Test set
1	MLP 60–5-1 BFGS17 Exp Ident	0.999	0.705
2	MLP 24–5-1 BFGS18 Tanh Tanh	0.999	0.905
3	MLP 13–3-1 BFGS26 Tanh Ident	0.977	0.975

MLP-multilayer perceptron; k-m-1-the number of input, hidden and output neurons; BFGSN-an algorithm for finding the minimum of the error function; Exp, Tanh, Ident-activation functions of the hidden and output layers of neurons, exponential, hyperbolic tangent, identical, respectively.

**Table 4:**
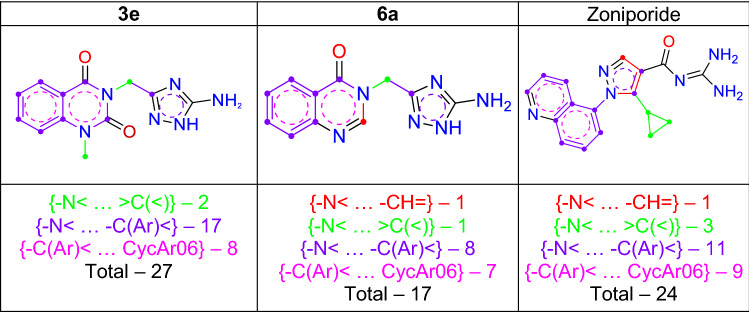
The entry of the identified pharmacophore into the structures of the new most active NHE-1 inhibitors and zoniporide.

Despite that zoniporide was excluded from the training set, the pharmacophore model obtained correctly reproduce its pharmacophore features. Therefore, we assume that the pharmacophore is valid and can be employed to design novel potent NHE-1inhibitors.

### Anti-inflammatory activity

**Figure 4:**
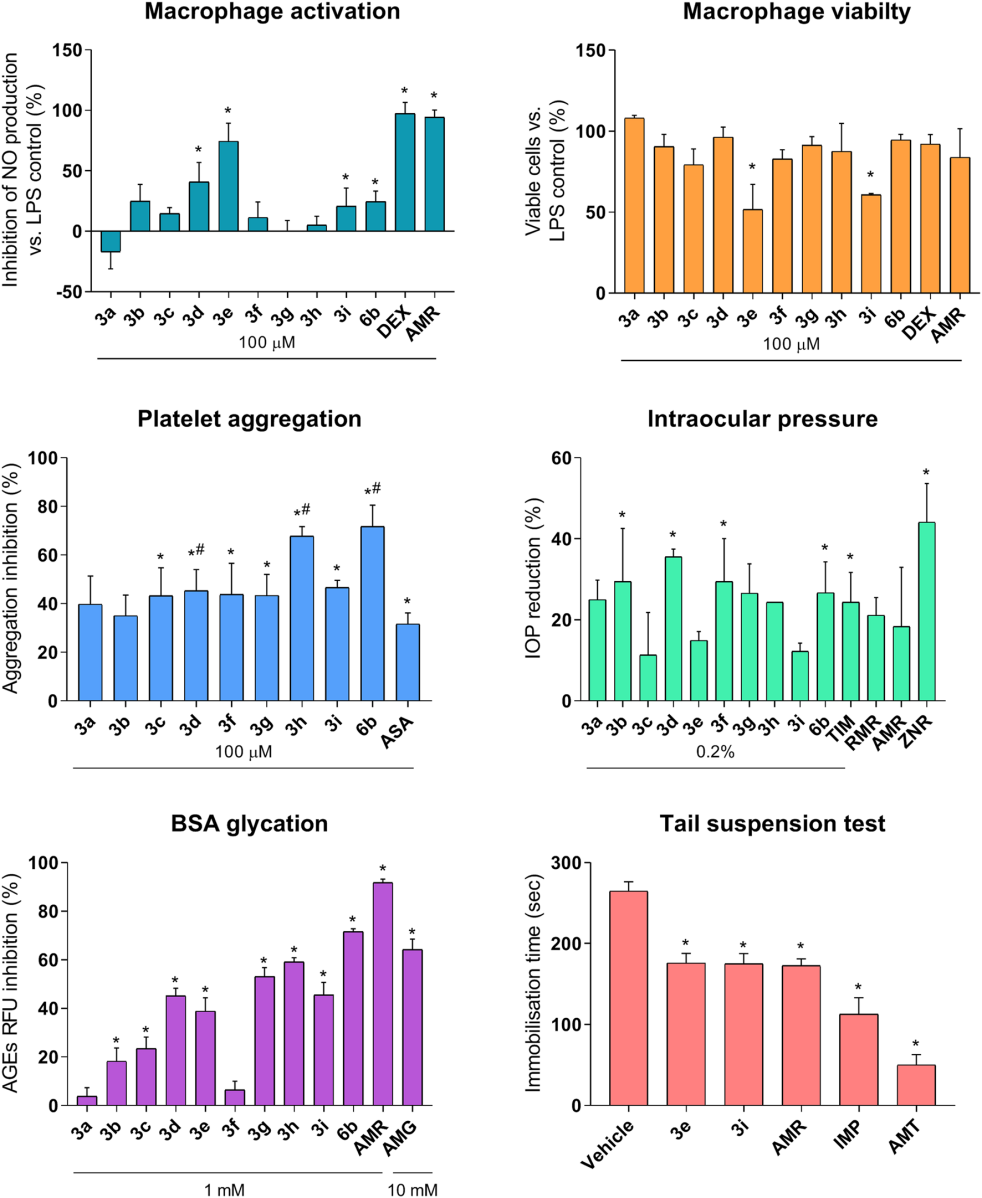
Pharmacological evaluation of the target compounds. Data are shown as mean ± SD. Statistical significance: *p < 0,05 vs. negative control; ^#^*p* < 0,05 vs. ASA (1-way ANOVA). *AMR* amiloride, *RMR* rimeporide, *ZNR* zoniporide, *TIM*-timolol, *DEX*-dexamethasone, *ASA*-acetylsalicylic acid, *AMG*-aminoguanidine, *IMP*-imipramine, *AMT*-amitriptyline.

**Figure 5:**
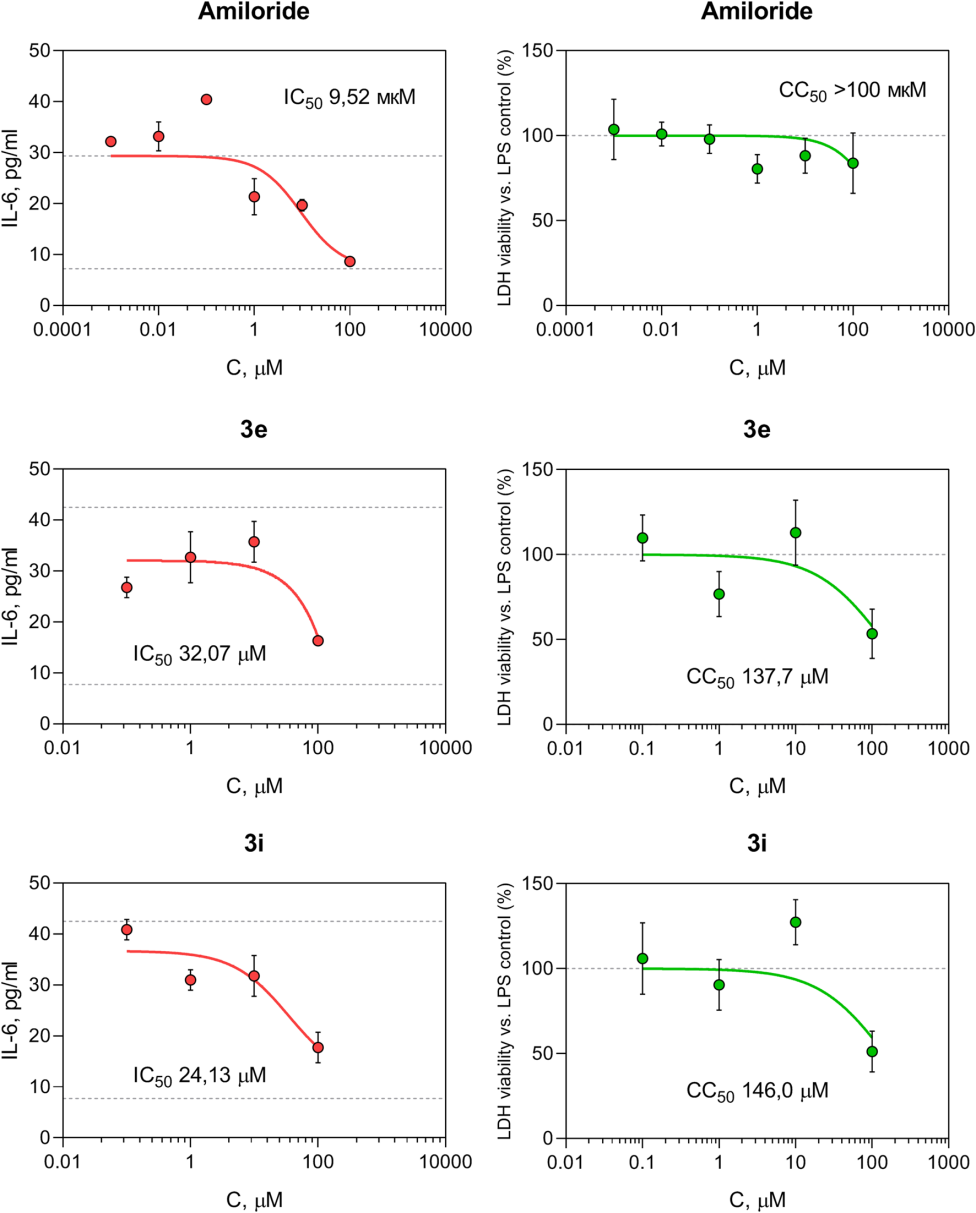
Lead compounds prevent IL-6 secretion from LPS-stimulated murine macrophages.

As a next step of the study target compounds were pharmacologically profiled for NHE-1 associated activities. A study of the anti-inflammatory activity was carried out on primary peritoneal macrophages of C57BL/6J mice (Fig. 4). Three compounds (**3d**, **3e**, **6b**) were found to statistically significantly inhibit the synthesis of nitric oxide at a concentration of 100 μM in the absence of a statistically significant effect on cell viability. The secretion of interleukin 6 was suppressed by four compounds – **3b**, **3c**, **3e**, **3i**. After a concentration-response validation study (Fig. 5), the lead compound **3i** was identified, which inhibits the synthesis of IL-6 with an IC_50_ of 24.13 μM and has moderate cytotoxicity (CC_50_ 146 μM), being inferior in activity to the reference drugs dexamethasone and amiloride.

### Antiplatelet activity

In addition, it was shown that the majority of them exhibits pronounced antiplatelet activity (Fig. 3). Unfortunately, the most active NHE-1 inhibitors **3e** and **6a** were insoluble under assay conditions. Four compounds (**6b**, **3a**, **3f** and **3h**) exceeded the activity of the reference drug, acetylsalicylic acid. Noteworthy, cyclic guanidine analogs again proved to be more active than compounds with acyclic guanidine side chains. Influence of *N*^1^ substituent on antiplatelet activity appears to be as follows: no substituent (**6b**) or methyl (**3a**, **3f**) > allyl (**3h**) > benzyl (**3c**, **3i**). Neither bromine at *C*^6^, nor methyl in the linker region (R^3^) had a definite effect on the activity.

### IOP-lowering activity

Effect on intraocular pressure (IOP) was studied on albino outbred rats. Two compounds (**3d**, **3e**) exhibit statistically significant activity, though the effect size is negligible for **3e**, while **3d** is comparable to zoniporide and superior to timolol. The complex anatomical structure of the eye presents a barrier for local drug delivery, which might explain a lack of correlation between NHE-1 inhibitory (and other cell-based assays) and IOP-reducing activities. Interestingly, the most active compound **3d** is also the most lipophilic one among the identified NHE-1 inhibitors. This is in line with several studies reporting that drug lipophilicity expressed as logD enhances both corneal and conjunctival permeability^26,27^.

### Antiglycating activity

Additionally, we have found that several compounds can effectively prevent the formation of advanced glycation products from bovine serum albumin and glucose. The most active compounds **6b** showed better potency than reference antiglycating drug aminoguanidine (IC_50_s 506.3 and 4521.2 µM, respectively). Since investigated compounds share similar nucleophilic centers and chelating abilities, which usually mediate antiglycating properties^28,29^, we were unable to rationalize SAR in this case. Presumably, higher activity is associated with the accessibility of the primary amine group of hydrazine (amiloride). Acyclic guanidine group instead of hydrazine is associated with lower activity^30^.

### Antidepressant activity

Suspension by the tail test was used to assess the antidepressant activity of lead compounds. It was found that compounds **3e** and **3i** exhibit antidepressant activity similar to amiloride^7^, although weaker than comparison drugs imipramine and amitriptyline.

## Discussion

NHE-1 inhibitors are experimentally and clinically validated pharmacological agents especially as potential cardioprotective therapies, agents against glaucoma and other disorders associated with ischemia and reperfusion, cell proliferative disorders and diabetes^31,32^. Modulation of sodium-hydrogen exchange prevents intracellular Ca^2+^ mobilization ameliorating ischemia-reperfusion cellular damage and platelet activation, thus NHE-1 inhibitors have cardioprotective^33^, neuroprotective^34^ and anti-arrhythmic properties^35^. Recently, guanidinecarbonyl^36^ and (imidazol4-yl-carbonyl)guanidine^37^ derivatives have been described as potent NHE-1 inhibitors, however, their pharmacological evaluation was limited to cardioprotective effect.

Herein we report a novel series of guanidine-modified quinazoline s as NHE-1 inhibitors that also possess anti-inflammatory and antiglycating properties, both of them being important additional mechanisms of action in conditions of systemic inflammation and diabetes mellitus, which are in turn associated with increased cardiovascular risk and retinopathy. Compounds **3a**, **3d**, **3h** have activity comparable to the reference drug rimeporide, while **3e** and **6a** are more potent and comparable to zoniporide. NHE-1 inhibition was confirmed in follow-up cellular and animal studies, although lack of apparent correlation with NHE-1 inhibition is likely due to cellular permeability and uptake factors.

Identified active compounds prevent ADP-induced platelet aggregation, LPS-induced macrophage activation, reduce intraocular pressure and also demonstrated some antidepressant potential, probably via amelioration of microglia activation^5^. We have identified NHE-1 inhibitors **3e** and **3i** to prevent LPS-induced macrophage polarization and IL-6 secretion in micromolar range with 4-6 selectivity over cytotoxicity. Along with this, **3e** and **3i** possess antidepressant activity comparable with amiloride. ADP-induced platelet aggregation was most effectively inhibited by compounds **3d**, **3h** and **6b**, which exceeded acetylsalicylic acid. NHE-1 inhibitor **3d** was also found to markedly reduce intraocular pressure. In conclusion, we posit that the identified pharmacophore may serve as a viable starting point for development of novel NHE-1 inhibitors endowed with valuable pharmacological activities. Further efforts will be undertaken to optimize the structure and potency of quinazoline-derived NHE-1 inhibitors.

## Methods

All reagents were procured from Panreac and Acros Organics at the highest grade available, and they were used without further purification. Anhydrous DMF was purchased from Sigma-Aldrich Co. Thin-layer chromatography (TLC) was performed on Merck TLC Silica gel 60 F254 plates by eluting with ethyl acetate or ethanol that was developed using a VL-6.LC UV lamp (Vilber). Yields refer to spectroscopically (^1^H and ^13^C NMR) homogeneous materials. The melting points were determined in glass capillaries on a Mel-Temp 3.0 apparatus (Laboratory Devices Inc., USA). The NMR spectra were recorded using Bruker Avance 400 (400 MHz for ^1^H and 100 MHz for ^13^C) spectrometer in DMSO-*d*_6_ with tetramethylsilane used as an internal standard. IR spectra were recorded on an FSM-1201 IR Fourier spectrometer (Russia) in KBr tablets.

### General procedure for synthesizing ester quinazolin-2,4(1*H*,3*H*)-dione derivatives (2a-f)

A mixture of N^1^-substituted quinazolin-2,4(1*H*,3*H*)-dione **1** (20.0 mmol), ester of bromoacetic or 2-bromopropionic acid (21.0 mmol), and K_2_CO_3_ (7.0 g, 50.6 mmol) was stirred in a DMF solution (100 mL) at room temperature for 24 h. The reaction mass was filtered, evaporated to dryness in vacuo; the residue was treated with water (100 mL); the solid residue was filtered off, dried at room temperature, and recrystallized from ethyl acetate.

Esters of quinazolin-4(3*H*)-one derivatives **5a-c** were synthesized similarly.

#### Benzyl (1-methyl-2,4-dioxo-1,4-dihydroquinazolin-3(2*H*)-yl)acetate (2a)

White solid (EtOAc); mp 152-154 °C; ^1^H NMR (DMSO-d_6_, 400 MHz) δ 8.06 (1H, d, *J* = 8 Hz, H-5), 7.71 (1H, t, *J* = 8 Hz, H-7), 7.39 (1H, d, *J* = 8 Hz, H-8), 7.32-7.37 (5H, m, Ph), 7.29 (1H, t, *J* = 8 Hz, H-6), 5.21 (2H, s, NCH_2_C(O)), 5.05 (2H, s, CH_2_O), 3.30 (3H, s, CH_3_); ^13^C NMR (DMSO-d_6_, 100 MHz) δ 168.06, 161.00, 150.54, 139.44, 135.49, 135.17, 128.37, 128.12, 127.90, 127.86, 123.04, 114.58, 114.26, 66.55, 44.76, 28.02.

#### Benzyl 2-[2,4-dioxo-1-(prop-2-en-1-yl)-1,4-dihydroquinazolin-3(2*H*)-yl]propanoate (2b)

White solid (EtOAc); mp 95-97 °C; ^1^H NMR (DMSO-d_6_, 400 MHz) δ 8.08 (1H, d, *J* = 8 Hz, H-5), 7.75 (1H, t, *J* = 8 Hz, H-7), 7.57 (1H, s, H-8), 7.23-7.33 (6H, m, H-6, Ph), 5.78-5.86 (1H, m, CH=), 5.60-5.62 (1H, m, CH), 5.05-5.15 (2H, m, =CH_2_), 4.52 (2H, s, CH_2_), 1.58 (3H, d, *J* = 7 Hz, CH_3_); ^13^C NMR (DMSO-d_6_, 100 MHz) δ 169.48, 160.42, 149.36, 139.23, 135.65, 135,54, 132.15, 128.42, 128.25, 127.94, 127.79, 123.15, 116.58, 114.87, 114.02, 66.32, 52.58, 42.88, 14.03.

#### Benzyl (1-benzyl-2,4-dioxo-1,4-dihydroquinazolin-3(2*H*)-yl)acetate (2c)

White solid (EtOAc); mp 142-144 °C; ^1^H NMR (DMSO-d_6_, 400 MHz) δ 8.08 (1H, d, *J* = 8 Hz, H-5), 7.67 (1H, t, *J* = 8 Hz, H-7), 7.20-7.39 (12H, m, H-6, H-8, Ph, Ph), 5.39 (2H, s, NCH_2_C(O)), 5.21 (2H, s, CH_2_O), 4.88 (2H, s, CH_2_); ^13^C NMR (DMSO-d_6_, 100 MHz) δ 168.00, 160.79, 150.58, 139.52, 136.02, 135.75, 135.60, 128.75, 128.50, 128.22, 128.19, 127.98, 127.39, 126.45, 123.36, 115.25, 114.72, 66.55, 46.31, 42.65.

#### Benzyl 2-(1-methyl-2,4-dioxo-1,4-dihydroquinazolin-3(2*H*)-yl)propanoate (2d)

White solid (EtOAc); mp 100-103 °C; ^1^H NMR (DMSO-d_6_, 400 MHz) δ 8.07 (1H, d, *J* = 8 Hz, H-5), 7.73 (1H, t, *J* = 8 Hz, H-7), 7.53 (1H, s, H-8), 7.23-7.32 (6H, m, H-6, Ph), 5.60-5.64 (1H, m, CH), 5.07-5.21 (2H, m, CH_2_), 3.28 (3H, c, NCH_3_), 1.59 (3H, d, *J* = 7 Hz, CH_3_); ^13^C NMR (DMSO-d_6_, 100 MHz) δ 169.51, 160.96, 149.83, 139.06, 135.76, 135.33, 128.25, 127.97, 127.73, 123.02, 114.85, 113.91, 66.21, 52.58, 27.81, 14.04.

#### Benzyl (4-oxoquinazolin-3(4*H*)-yl)acetate (2e)

White solid (Me_2_CHOH); mp 116-117 °C; ^1^H NMR (DMSO-d_6_, 400 MHz) δ 8.41 (1H, s, H-2), 8.16 (1H, d, *J* = 8 Hz, H-5), 7.85 (1H, t, *J* = 8 Hz, H-7), 7.71 (1H, d, *J* = 8 Hz, H-8), 7.57 (1H, t, *J* = 8 Hz, H-6), 7.30-7.38 (5H, m, Ph), 5.22 (2H, s, NCH_2_C(O)), 4.92 (2H, s, CH_2_O); ^13^C NMR (DMSO-d_6_, 100 MHz) δ 168.24, 160.52, 148.24, 148.21, 135.78, 135.04, 128.79, 128.56, 128.29, 127.70, 127.65, 126.35, 121.57, 66.93, 47.63.

#### Propan-2-yl (6-bromo-4-oxoquinazolin-3(4*H*)-yl)acetate (2f)

Light yellow solid (EtOAc); mp 114-116 °C; ^1^H NMR (DMSO-d_6_, 400 MHz) δ 8.41 (1H, s, H-2), 8.18 (1H, d, *J* = 2 Hz, H-5), 7.93 (1H, dd, *J* = 9 Hz, 2 Hz, H-7), 7.62 (1H, d, J = 9 Hz, H-8), 4.97 (1H, m, *J* = 6 Hz, CH), 4.81 (2H, s, NCH_2_C(O)), 1.20 (6H, d, *J* = 6 Hz, CH_3_); ^13^C NMR (DMSO-d_6_, 100 MHz) δ 170.56, 162.37, 151.83, 150.26, 140.79, 133.03, 131.45, 126.13, 123.16, 72.54, 50.86, 24.82.

### General procedure for synthesizing guanidine quinazolin-2,4(1*H*,3*H*)-dione derivatives (3a-d)

A mixture of N^1^-substituted quinazolin-2,4(1*H*,3*H*)-dione ester derivative (5.0 mmol), guanidine hydrochloride (0.5 g, 5.2 mmol), and KOH (0.3 g, 5.4 mmol) was refluxed in 95% ethanol solution (25 mL) for 10 min. The hot reaction mass was filtered and cooled. The solid residue was filtered off, dried at room temperature, and recrystallized from ethanol.

#### *N*-Carbamimidoyl-2-(1-methyl-2,4-dioxo-1,4-dihydroquinazolin-3(2*H*)-yl)acetamide (3a)

White solid (EtOH); mp 266–269 °C; ^1^H NMR (DMSO-d_6_, 400 MHz) δ 8.01 (1H, d, *J* = 7.5 Hz, H-5), 7.60–7.70 (5H, m, H-7, NH), 7.22 (1H, t, *J* = 7.5 Hz, H-6), 7.17 (1H, d, *J* = 8 Hz, H-8), 4.43 (2H, s, NCH_2_C(O)), 3.31 (3H, s, CH_3_); ^13^C NMR (DMSO-d_6_, 100 MHz) δ 171.01, 161.32, 158.80, 150.45, 140.44, 134.83, 127.40, 122.15, 114.77, 114.29, 47.08, 27.95; HRMS-ESI: MH^+^, found: C_12_H_13_N_5_O_3_ [M + H]^+^ 276.1091, requires: 276.1097.

#### *N*-Carbamimidoyl-2-(1-methyl-2,4-dioxo-1,4-dihydroquinazolin-3(2*H*)-yl)propanamide (3b)

White solid (EtOH); mp 216-219 °C; ^1^H NMR (DMSO-d_6_, 400 MHz) δ 8.03 (1H, d, *J* = 8 Hz, H-5), 7.65 (1H, t, *J* = 8 Hz, H-7), 7.27 (1H, d, *J* = 7.5 Hz, H-8), 7.21 (1H, t, *J* = 7.5 Hz, H-6), 5,42-5,44 (1H, m, CH), 3.32 (3H, s, NCH_3_), 1.49 (3H, d, *J* = 7.5 Hz, CH_3_); ^13^C NMR (DMSO-d_6_, 100 MHz) δ 173.48, 161.28, 158.69, 150.53, 139.35, 134.25, 127.66, 122.02, 115.80, 114.85, 53.96, 28.13, 15.18; HRMS-ESI: MH^+^, found: C_13_H_15_N_5_O_3_ [M+H]^+^ 290.1248, requires: 290.1253; found: [M+Na]^+^ 312.1067, requires: 312.1073.

#### 2-(1-Benzyl-2,4-dioxo-1,4-dihydroquinazolin-3(2*H*)-yl)-*N*-carbamimidoylacetamide (3c)

White solid (EtOH); mp 180–183 °C; ^1^H NMR (DMSO-d_6_, 400 MHz) δ 8.06 (1H, d, *J* = 8 Hz, H-5), 7.61–7.67 (5H, m, H-7, NH), 7.21–7.33 (7H, m, H-6, H-8, Ph), 5.38 (2H, s, NCH_2_), 4.38 (2H, s, NCH_2_C(O)); ^13^C NMR (DMSO-d_6_, 100 MHz) δ 171.25, 160.79, 158.80, 150.94, 139.47, 136.34, 134.93, 128.60, 127.99, 127.15, 126.50, 122.68, 115.28, 114.75, 45.96, 44.99; HRMS-ESI: MH^+^, found: C_18_H_17_N_5_O_3_ [M + H]^+^ 352.1404, requires: 352.1410; found: [M + Na]^+^ 374.1224, requires: 374.1229.

#### 2-(6-Bromo-1-methyl-2,4-dioxo-1,4-dihydroquinazolin-3(2*H*)-yl)-*N*-carbamimidoylacetamide (3d)

Light yellow solid (EtOH); mp 214–217; ^1^H NMR (DMSO-d_6_, 400 MHz) δ 8.03 (1H, s, H-5), 7.62 (1H, d, *J* = 9 Hz, H-7), 7.55 (4H, s, NH), 7.14 (1H, d, *J* = 9 Hz, H-8), 4.41 (2H, s, NCH_2_C(O)), 3.30 (3H, s, CH3); ^13^C NMR (DMSO-d_6_, 100 MHz) δ 170.62, 160.19, 158.72, 150.16, 139.70, 137.27, 129.17, 117.55, 115.99, 113.98, 47.22, 28.16; HRMS-ESI: MH^+^, found: C_12_H_12_BrN_5_O_3_ [M + H]^+^ 354.0196, requires: 354.0202; found: [M + Na]^+^ 376.0016, requires: 376.0021.

### General procedure for synthesizing 5-amino-1,2,4-triazole quinazolin-2,4(1*H*,3*H*)-dione derivatives (3e-i)

A mixture of *N*^1^-substituted quinazolin-2,4(1*H*,3*H*)-dione ester derivative (5.0 mmol), aminoguanidine carbonate (0.75 g, 5.5 mmol), and KOH (0.6 g, 10.7 mmol) was refluxed in 95% ethanol solution (25 mL) for 1 h. The hot reaction mass was filtered and cooled. The solid residue was filtered off, dried at room temperature, and recrystallized from ethanol. Quinazolin-4(3*H*)-one derivatives **6a-b** were synthesized similarly.

#### 3-[(5-Amino-4*H*-1,2,4-triazol-3-yl)methyl]-1-methylquinazoline-2,4(1*H*,3*H*)-dione (3e)

White solid (EtOH); mp 351-354 °C; ^1^H NMR (DMSO-d_6_, 400 MHz) δ 8.01 (1H, d, J = 8 Hz, H-5), 7.66 (1H, t, *J* = 8 Hz, H-7), 7.21 (1H, t, *J* = 7.5 Hz, H-6), 7.16 (1H, d, *J* = 8.5 Hz, H-8), 4.36 (2H, s, NCH_2_), 3.31 (3H, s, CH_3_); ^13^C NMR (DMSO-d_6_, 100 MHz) δ 168.97, 161.43, 150.47, 140.70, 134.70, 127.26, 121.97, 115.09, 114.28, 47.64, 27.95; HRMS-ESI: MH^+^, found: C_12_H_12_N_6_O_2_ [M+H]^+^ 273.1095, requires: 273.1100; found: [M+Na]^+^ 295.0914, requires: 295.0919.

#### 3-[1-(5-Amino-4*H*-1,2,4-triazol-3-yl)ethyl]-1-methylquinazoline-2,4(1*H*,3*H*)-dione (3f)

White solid (EtOH, solvate 1:1); mp 91-93 °C; ^1^H NMR (DMSO-d_6_, 400 MHz) δ 8.06 (1H, d, *J* = 8 Hz, H-5), 7.75 (1H, t, *J* = 7.5 Hz, H-7), 7.53 (1H, t, *J* = 7.5 Hz, H-6), 7.30 (1H, d, *J* = 7.5 Hz, H-8), 5.50-5.53 (1H, m, NCH), 4.03-4.18 (2H, CH_2_, EtOH), 3.28 (3H, s, NCH_3_), 1.54 (3H, d, *J* = 7 Hz, CH_3_), 1.10 (3H, t, *J* = 7 Hz, CH_3_, EtOH); ^13^C NMR (DMSO-d_6_, 100 MHz) δ 169.53, 161.00, 149.76, 139.08, 135.32, 128.24, 122.99, 114.84, 113.91, 60.76, 52.45, 27.81, 13.95, 13.86; HRMS-ESI: MH^+^, found: C_13_H_14_N_6_O_2_ [M+H]^+^ 287.1251, requires: 287.1256; found: [M+Na]^+^ 309.1070, requires: 309.1076.

#### 3-[(5-Amino-4*H*-1,2,4-triazol-3-yl)methyl]-1-(prop-2-en-1-yl)quinazoline-2,4(1*H*,3*H*)-dione (3g)

White solid (EtOH); mp 275-277 °C; ^1^H NMR (DMSO-d_6_, 400 MHz) δ 8.01 (1H, d, *J* = 8 Hz, H-5), 7.67 (1H, t, *J* = 8 Hz, H-7), 7.22 (1H, t, *J* = 7.5 Hz, H-6), 7.17 (1H, d, *J* = 8.5 Hz, H-8), 5.83-5.92 (1H, m, CH=), 5.09-5.15 (2H, m, CH_2_=), 4.55 (2H, d, *J* = 5.5 Hz, CH_2_), 4.35 (2H, s, NCH_2_); ^13^C NMR (DMSO-d_6_, 100 MHz) δ 168.55, 160.94, 150.01, 140.92, 134.81, 132.6,5, 127.35, 122.02, 116.56, 115.26, 114.31, 47.73, 42.93; HRMS-ESI: MH^+^, found: C_14_H_14_N_6_O_2_ [M+H]^+^ 299.1251, requires: 299.1256; found: [M+Na]^+^ 321.1070, requires: 321.1076.

#### 3-[1-(5-Amino-4*H*-1,2,4-triazol-3-yl)ethyl]-1-(prop-2-en-1-yl)quinazoline-2,4(1*H*,3*H*)-dione (3h)

White solid (EtOH, solvate 1:1); mp 116–118; ^1^H NMR (DMSO-d_6_, 400 MHz) δ 8.07 (1H, d, *J* = 8 Hz, H-5), 7.77 (1H, t, *J* = 8 Hz, H-7), 7.54 (1H, d, *J* = 8 Hz, H-8), 7.31 (1H, t, *J* = 7.5 Hz, H-6), 5.82–5.91 (1H, m, CH =), 5.47–5.51 (1H, m, NCH), 5.03–5.12 (2H, m, CH_2_ =), 4.48–4.56 (2H, m, NCH_2_), 4.00–4.15 (2H, m, CH_2_, EtOH), 1.53 (3H, d, *J* = 7 Hz, CH_3_), 1.09 (3H, t, *J* = 7 Hz, CH_3_, EtOH); ^13^C NMR (DMSO-d_6_, 100 MHz) δ 169.44, 160.44, 149,26, 139.27, 135.52, 132.17, 128.38, 123.09, 116.29, 114.84, 113.98, 60.76, 52.52, 42.78, 13.94, 13.81; HRMS-ESI: MH^+^, found: C_15_H_16_N_6_O_2_ [M + H]^+^ 313.1408, requires: 313.1413; found: [M + Na]^+^ 335.1227, requires: 335.1232.

#### 3-[(5-Amino-4*H*-1,2,4-triazol-3-yl)methyl]-1-benzylquinazoline-2,4(1*H*,3*H*)-dione (3i)

White solid (EtOH); mp 262-265 °C; ^1^H NMR (DMSO-d_6_, 400 MHz) δ 8.04 (1H, d, *J* = 8 Hz, H-5), 7.61 (1H, t, *J* = 8 Hz, H-7), 7.20-7.32 (7H, m, H-6, H-8, Ph), 5.37 (2H, s, NCH_2_), 4.33 (2H, s, CH_2_); ^13^C NMR (DMSO-d_6_, 100 MHz) δ 169.48, 166.46, 160.87, 151.03, 139.48, 136.43, 134.79, 128.60, 127.96, 127.13, 126.49, 122.56, 115.39, 114.76, 45.98, 45.56; HRMS-ESI: MH^+^, found: C_18_H_16_N_6_O_2_ [M+H]^+^ 349.1408, requires: 349.1413; found: [M+Na]^+^ 371.1227, requires: 371.1232.

#### 3-[(5-Amino-4*H*-1,2,4-triazol-3-yl)methyl]quinazolin-4(3*H*)-one (6a)

White solid (EtOH); mp 311–314 °C; ^1^H NMR (DMSO-d_6_, 400 MHz) δ 8.19 (1H, s, H-2), 8.12 (1H, d, *J* = 8 Hz, H-5), 7.78 (1H, t, *J* = 7.5 Hz, H-7), 7.64 (1H, d, *J* = 8 Hz, H-8), 7.49 (1H, t, *J* = 7.5 Hz, H-6), 4.32 (2H, s, CH_2_); ^13^C NMR (DMSO-d_6_, 100 MHz) δ 169.35, 160.15, 149.27, 148.11, 133.74, 126.91, 126.40, 125.98, 121.79, 49.32; HRMS-ESI: MH^+^, found: C_18_H_17_N_5_O_3_ [M + H]^+^ 352.1404, requires: 352.1410; found: [M + Na]^+^ 374.1224, requires: 374.1229.

#### 3-[(5-Amino-1*H*-1,2,4-triazol-3-yl)methyl]-6-bromoquinazolin-4(3*H*)-one (6b)

Light yellow solid (EtOH); mp 321-324; ^1^H NMR (DMSO-d_6_, 400 MHz) δ 8.21 (1H, s, H-2), 8.20 (1H, s, H-5), 7.89 (1H, d, *J* = 8.5 Hz, H-7), 7.59 (1H, d, *J* = 8 Hz, H-8), 4.32 (2H, s, CH_2_); ^13^C NMR (DMSO-d_6_, 100 MHz) δ 168.97, 168.94, 159.05, 149,85, 147.11, 136.57, 129.39, 128.09, 123.33, 118.82, 49.48; HRMS-ESI: MH^+^, found: C_11_H_9_BrN_6_O [M+H]^+^ 321.0094, requires: 321.0099; found: [M+Na]^+^ 342.9913, requires: 342.9919.

### Compound preparation

Test compounds were dissolved in 99% DMSO (stock concentration 40 mM) and stored at –25 °C. If sediment or opalescence was detected, 5% Tween 20 (Merck) was added. Serial dilutions were prepared *ex tempore* in a media suitable for the particular study. Final concentration in samples: DMSO <0.25%, Tween 20 <0.025% (were added to control samples in equal concentrations).

### Animals

All procedures with animals in the study were carried out under the generally accepted ethical standards for the manipulations on animals adopted by the European Convention for the Protection of Vertebrate Animals used for Experimental and Other Scientific Purposes (1986) and taking into account the International Recommendations of the European Convention for the Protection of Vertebrate Animals used for Experimental research (1997). The study was approved by the Local Ethics Committee of the Volgograd State Medical University (registration No. IRB 00005839 IORG 0004900, OHRP), Certificate No. 2021/056, 15.06.2021. All sections of this study adhere to the ARRIVE Guidelines for reporting animal research^38^.

### NHE-1 inhibition assay

Evaluation of NHE-1 inhibition was carried out on rabbit platelets by the known method^39,40^. The experiments were carried out on 15 male rabbits weighing 3.0-4.0 kg. Platelet-rich plasma (PRP) was obtained by centrifuging blood with 3.8% sodium citrate (1:10) at 1000 rpm for 12 min (Multi centrifuge CM 6M, Latvia). Platelet shapeshifting due to acidification was followed with a laser aggregometer BIOLA-220 LA (Russia). Test compounds (10 μl, 10 nM final concentration) were added to 200 μl of PRP 5 min in a cuvette before the addition of sodium propionate solution, incubated with constant stirring using a magnetic stirrer (800 rpm, 37 °C). To control samples for NHE-1 activation, a buffer solution containing sodium propionate was added to 200 μL of PRP (600 μL, 135 mM sodium propionate, 20 mM HEPES, 1 mM CaCI_2_, 1 mM MgCI_2_, 10 mM glucose; pH 6.7). The change in light transmission at pH 7.4, 37 °C was monitored in a Krebs solution (600 μL, 120 mM NaCl, 4.8 mM KCl, 1.2 mM KH_2_PO_4_, 2.5 mM MgSO_4_, 25 mM NaHCO_3_, 2.6 mM CaCl_2_, 5.4 mM glucose; pH 7.4). Test compounds were added to a final concentration of 10 nM to the cuvette into 200 μl of PRP 5 min before adding the sodium propionate solution, incubated with constant stirring using a magnetic stirrer (800 rpm, 37 °C). NHE-1 inhibitors zoniporide, rimeporide, amiloride were used as reference drugs. Assays were run in 6 independent series.

### Pharmacophore modeling

Structures of 13 target compound were characterized with a matrix of *QL*-descriptors of the 2nd rank of the 5th type using IT Microcosm system^41^. *QL*-descriptor matrix and experimental values of NHE-1 inhibitory activity activity for these substances served as an initial training set. We used a two-layer perceptron with a bottleneck MLP *k*-*m*-1 for neural network modeling of regression dependence, where the number of input neurons *k* >> *m* is the number of hidden neurons; calculations were performed in the Statistica program^42^. Iterative training of networks was performed with the division of the initial dataset into training and test sets in a ratio of 60/40% with an automatic selection of neural networks with high values of the correlation coefficients. Training of each of the 500 neural networks involved random selection of training and test sets to minimize possible bias. For the best performing neural network, sensitivity analysis (*Sens*) of input neurons was performed, low-sensitivity neurons were removed, and iterative neural network modeling was performed. In the best neural network, the most sensitive input neurons were identified. By superposition of the found significant *QL*-descriptors, a pharmacophore was formed that defines a high level of NHE-1 inhibitory activity of the tested compounds. Zoniporide structure was excluded from training sets but was used to validate the model.

### Platelet aggregation assay

Functional activity of platelets was determined on a two-channel laser analyzer of platelet aggregation "BIOLA-220 LA" (Russia) as described previously^43^. The experiments were carried out on 6 male rabbits weighing 3.5–4 kg. To prepare platelet-rich plasma (PRP) venous blood was taken from the ear marginal vein of a rabbit, stabilized with a 3.8% sodium citrate solution in a ratio of 9:1 and centrifuged for 10 min at 1500 rpm. PRP (300 μL) and a solution of the test compound at a concentration of 100 μM were sequentially introduced into the cell of the aggregometer. The samples were incubated in thermostated cells of the aggregometer at 37 °C for 5 min. To induce aggregation adenosine-5-diphosphoric acid (ADP, Sigma, USA) at a final concentration of 5 μM, was added to the cuvette. Acetylsalicylic acid (Shandong Xinhua Pharmaceutical Co., Ltd., China) was used as a reference drug. Experiments were run in 5 independent replicates.

### Isolation and treatment of peritoneal macrophages

Peritoneal macrophages (PM) were isolated from the peritoneal exudate of 30 male C57bl/6j mice. To accumulate PM, 1 ml of 3% peptone solution was injected intraperitoneally. After 3 days the mice were euthanized by cervical dislocation. Cells of peritoneal exudate were obtained by aseptically washing the abdominal cavity with 5 ml of sterile Hanks’s solution (+ 4-6 °C) without calcium and magnesium ions. The total number and viability of cells were assessed in a Goryaev counting chamber (Russia) with a 0.4% trypan blue staining (Sigma-Aldrich, USA). The cell concentration was adjusted to 1.0×10^6^ cells/ml in DMEM (Gibco) supplemented with 2 mM L-glutamine (Gibco), 10% heat-inactivated fetal bovine serum (BioClot, Germany), 100 U/ml penicillin and 100 mg/ml streptomycin (Gibco) and plated 200 μl/well in 96-well transparent plates (SPL Life Sciences Co., Ltd., Korea). It was left for 2 h at 37°C in a humidified atmosphere with 5% CO_2_, after which the wells were washed to remove non-adherent cells. After 24 hours of incubation, 20 μl of the supernatant was removed and 20 μl of solutions of test compounds were added 30 min before *E. coli* O127:B8 LPS (100 ng/ml final concentration). Experiments were run in 3 independent replicates.

### Assay of nitric oxide (NO)

The accumulation of nitrite anion (a stable end product of NO decomposition produced by iNOS) in supernatants was determined using a standard Griess reagent. Briefly, 50 μl of supernatants collected 22 hours after incubation of PM with test and control compounds were mixed with 50 μl of 1% sulfonamide in 2.5% H_3_PO_4_ and 50 μl of 0.1% *N*-(1-naphthyl) ethylenediamine in 2,5% H_3_PO_4_. After incubation at 23 °C for 10 min in an orbital shaker, the optical density was determined at a wavelength of 550 nm with a microplate reader Infinite M200 PRO (Tecan, Austria).

### Assay of interleukin-6 (IL-6)

Cell supernatant was collected and centrifuged at 1000 g for 20 min in a 2-16PK Sigma centrifuge (Germany). The concentration of IL-6 was determined by ELISA using a commercial kit (Cloud-clone ELISA kit) with a microplate reader Infinite M200 PRO (Tecan, Austria).

### Cytotoxicity study

The activity of lactate dehydrogenase (LDH) in a cell culture medium served as a marker of membrane permeability and cell death. Aliquotes of supernatants were taken after 24 h of inoculation with test compounds, mixed with 250 μl of 0.194 nM NADH solution in 54 mM phosphate buffered saline (pH 7.5). Then, 25 μl of a 6.48 mM pyruvate solution was added to the mixture. The optical density was followed at a wavelength of 340 nm for 20 min. Conversion of optical density into cell viability was carried out according to a standard curve (DMSO-treated cells as 100% and 0.01% Triton X-100-treated cells as 0% viable cells).

### Intraocular pressure studies

The experiments were carried out on 75 adult outbred rats of both sexes, which were kept in standard cages at a temperature of 25 °C and a standard light regime. Animals were randomly assigned to the control and experimental groups (n = 5). Before the start of the experiment, the rats had free access to food and water. Intraocular pressure (IOP) was measured with a TonoVet device (Finland)^44^ which measures IOP with a short touch of a small disposable tip in the center of the cornea, no corneal anesthesia is required. The study was carried out according to the published method^45^. One drop (50 μl) of a test 0.4% solution of the compound was instilled into the right eye, and deionized water was added to the left eye. IOP was measured in both eyes. The left eye, in turn, serves to assess the possible systemic exposure of the test compounds. Drugs with NHE-1-inhibitory activity were instilled in concentrations suggested in therapeutic practice - 0.2% zoniporide, 0.4% amiloride. IOP was measured at five time points (0, 1, 2, 3, and 4 hrs), where 0 hour is the baseline value. IOP-lowering activity was assessed as the maximum decrease in IOP from the initial values. At 9:00 AM, baseline IOP was measured in animals of all groups. Experiments were run in 5 independent replicates.

### Study of antiglycating activity

The glycation reaction was modeled using 1 mg/ml bovine serum albumin (Chemmed, Russia) and 0.36 M glucose (Vekton, Russia) in 50 mM phosphate buffer solution (PBS, pH 7.4) at 60 °C. After 24 hrs, albumin was precipitated using trichloroacetic acid (10% final concentration) and centrifugation (15 000 rpm, 4 min). The supernatant was removed by aspiration, and the residue was dissolved in 50 mM PBS (pH 10.5). Aliquots of 300 µl were transferred to a black flat-bottom 96-well microplate. AGE fluorescence was registered using Infinite M200 Pro (Tecan, Austria) microplate reader at excitation/emission wavelengths of 370/440 nm. Signals were normalized using blank samples containing BSA and the test compound in the appropriate concentration without the glucose. When normalized, the activity is expressed as the AGE fluorescence coefficient, determined by the formula:

$$Flu = 10^{\log_{10}{A} - \log_{10}{B} } - 1,$$

where *A* and *B* correspond to the absolute fluorescence values of the glucose-containing and mean glucose-free sample.

Activity percentage was calculated using the formula:

$$A_{\% } = \left( {1 - \frac{{Flu\left( {Sample} \right)}}{{Flu\left( {Control} \right)}}} \right) \times 100\%,$$

where *Flu(Sample)* and *Flu(Control)* correspond to the normalized fluorescence coefficients of experimental and control samples. Experiments were run in 5 independent replicates.

### Tail suspension test

The experiment was carried out on 36 male ICR mice weighing 22-25 g, divided into groups of 6 animals each under room lighting (300 lux). Tail suspension test^46^ was performed using the Panlab LE808 apparatus, suspended by the tail with a piece of adhesive tape (painless method), instinctively tries to free itself from an unpleasant situation, after unsuccessful attempts to escape, the animal begins to demonstrate the behavior of despair - immobilization. At the same time, it is believed that the severity of despair, determined by immobility, directly depends on depressive disorders in the subjects, and is significantly reduced when taking antidepressants. In experiments, test substances were administered intragastrically 30 min before the start of the test. Experimental groups received test compounds in an equimolar dose to the comparison drug amiloride. Animals of the remaining groups were treated with 2.6 mg/kg amiloride, 10 mg/kg amitriptyline, or 8 mg/kg imipramine. Distilled water was administered to the control group. The time of immobilization of the animals was recorded.

## Supplementary Information

Supplementary Information.
