# Supplementary material for: Synthesis and multifaceted pharmacological activity of novel quinazoline NHE-1 inhibitors
Source: Sci Rep. 2021 Dec 21;11:24380. doi: 10.1038/s41598-021-03722-w (PMC8692498; doi:10.1038/s41598-021-03722-w)

Synthesis and multifaceted pharmacological activity of novel quinazoline NHE-1 inhibitors

**Alexander Spasov**^1,2,+^**, Alexander Ozerov**^2,3,+^**, Pavel Vassiliev**^1,2,+^**, Vadim Kosolapov**^1,2,+^**, Natalia Gurova**^1,2^**, Aida Kucheryavenko**^1^**, Ludmila Naumenko**^1^**, Denis Babkov**^1,2,*,+^**, Viktor Sirotenko**^1,2^**, Alena Taran**^1,2^**, Roman Litvinov**^1,2^**, Alexander Borisov**^2^**, Vladlen Klochkov**^1^**, Darya Merezhkina**^1^**, Mikhail Miroshnikov**^1,2^**, Georgy Uskov**^1^ **and Nadezhda Ovsyankina**^1^

^1^Volgograd State Medical University, Department of Pharmacology & Bioinformatics, Volgograd, 400131, Russia

^2^Volgograd State Medical University, Scientific Center for Innovative Drugs, Volgograd, 400087, Russia

^3^Volgograd State Medical University, Department of Pharmaceutical & Toxicological Chemistry, Volgograd, 400131, Russia

^*^dababkov@volgmed.ru

**Supplementary data**

**
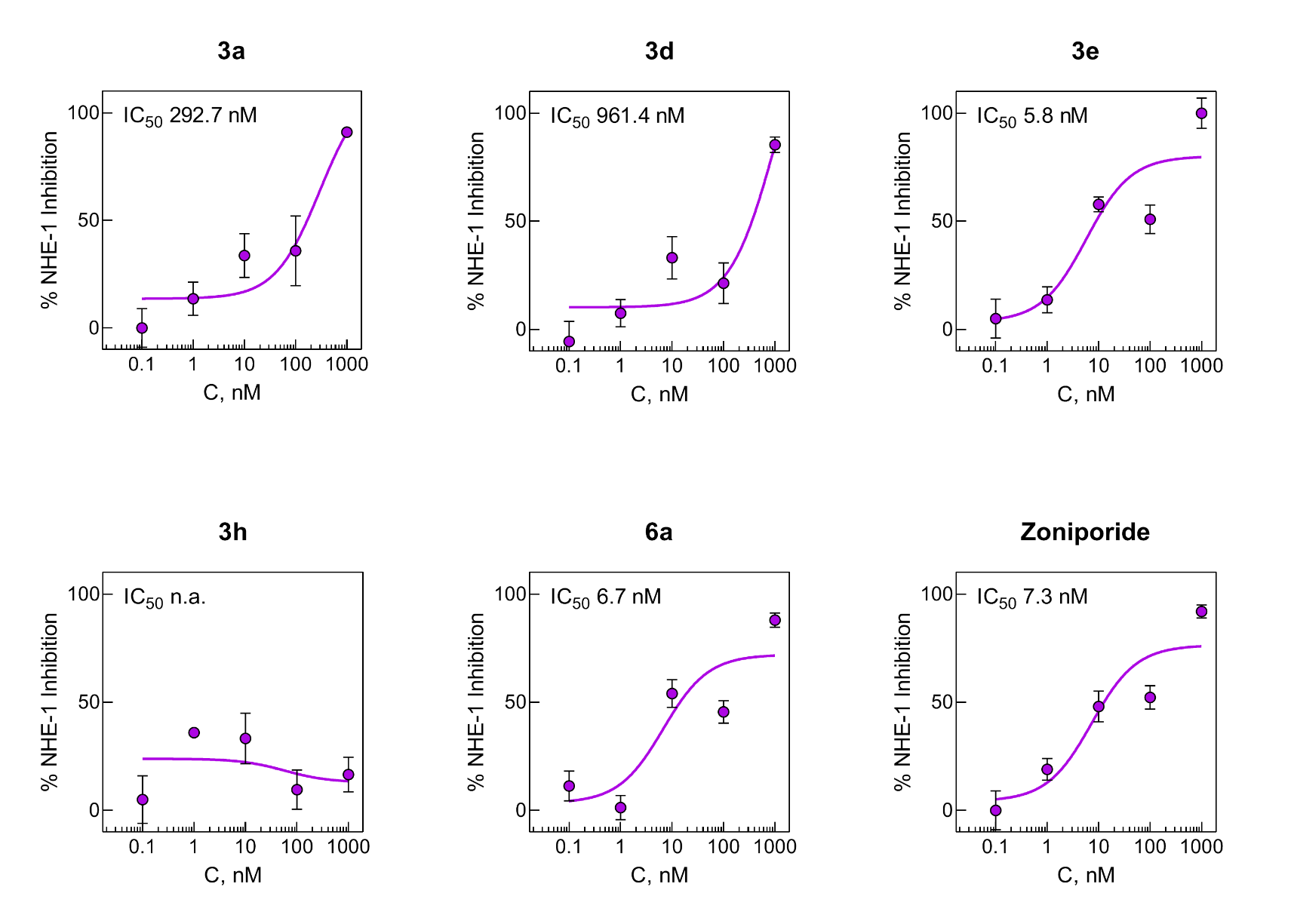
**

**Figure S1.** NHE-1 inhibition curves obtained for the most active compounds.

Data are shown as mean ±SD from 6 independent experiments.

**Copies of NMR ^1^H, ^13^C and IR spectra**

**^1^H NMR (DMSO-d_6_, 400 MHz): Benzyl (1-methyl-2,4-dioxo-1,4-dihydroquinazolin-3(2*H*)-yl)acetate (2a)**

**^13^C NMR (DMSO-d_6_, 100 MHz): Benzyl (1-methyl-2,4-dioxo-1,4-dihydroquinazolin-3(2*H*)-yl)acetate (2a)**

**^1^H NMR (DMSO-d_6_, 400 MHz): Benzyl 2-[2,4-dioxo-1-(prop-2-en-1-yl)-1,4-dihydroquinazolin-3(2*H*)-yl]propanoate (2b)**

**^13^C NMR (DMSO-d_6_, 100 MHz): Benzyl 2-[2,4-dioxo-1-(prop-2-en-1-yl)-1,4-dihydroquinazolin-3(2*H*)-yl]propanoate (2b)**

**^1^H NMR (DMSO-d_6_, 400 MHz): Benzyl (1-benzyl-2,4-dioxo-1,4-dihydroquinazolin-3(2*H*)-yl)acetate (2c)**

**^13^C NMR (DMSO-d_6_, 100 MHz): Benzyl (1-benzyl-2,4-dioxo-1,4-dihydroquinazolin-3(2*H*)-yl)acetate (2c)**

**^1^H NMR (DMSO-d_6_, 400 MHz): Benzyl 2-(1-methyl-2,4-dioxo-1,4-dihydroquinazolin-3(2*H*)-yl)propanoate (2d)**

**^13^C NMR (DMSO-d_6_, 100 MHz): Benzyl 2-(1-methyl-2,4-dioxo-1,4-dihydroquinazolin-3(2*H*)-yl)propanoate (2d)**

**^1^H NMR (DMSO-d_6_, 400 MHz): Benzyl (4-oxoquinazolin-3(4*H*)-yl)acetate (2e)**

**^13^C NMR (DMSO-d_6_, 100 MHz): Benzyl (4-oxoquinazolin-3(4*H*)-yl)acetate (2e)**

**^1^H NMR (DMSO-d_6_, 400 MHz): Propan-2-yl (6-bromo-4-oxoquinazolin-3(4*H*)-yl)acetate (2f)**

**^13^C NMR (DMSO-d_6_, 100 MHz): Propan-2-yl (6-bromo-4-oxoquinazolin-3(4*H*)-yl)acetate (2f)**

**^1^H NMR (DMSO-d_6_, 400 MHz): *N*-Carbamimidoyl-2-(1-methyl-2,4-dioxo-1,4-dihydroquinazolin-3(2*H*)-yl)acetamide (3a)**

**^13^C NMR (DMSO-d_6_, 100 MHz): *N*-Carbamimidoyl-2-(1-methyl-2,4-dioxo-1,4-dihydroquinazolin-3(2*H*)-yl)acetamide (3a)**

**IR-spectrum: *N*-Carbamimidoyl-2-(1-methyl-2,4-dioxo-1,4-dihydroquinazolin-3(2*H*)-yl)acetamide (3a)**

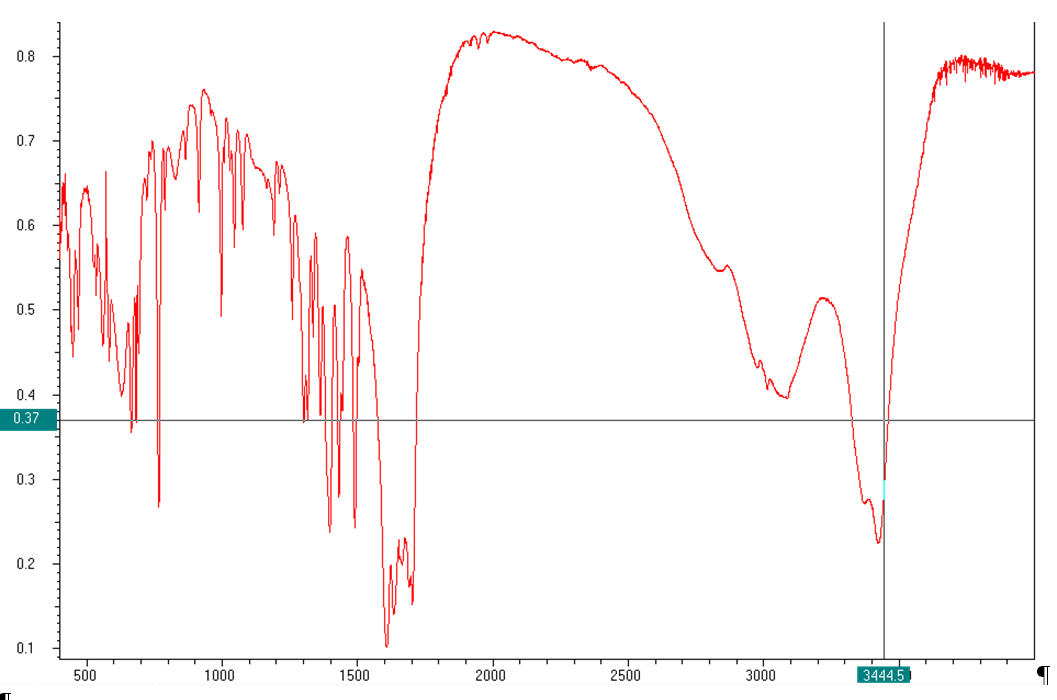


**^1^H NMR (DMSO-d_6_, 400 MHz): *N*-Carbamimidoyl-2-(1-methyl-2,4-dioxo-1,4-dihydroquinazolin-3(2*H*)-yl)propanamide (3b)**

**^13^C NMR (DMSO-d_6_, 100 MHz): *N*-Carbamimidoyl-2-(1-methyl-2,4-dioxo-1,4-dihydroquinazolin-3(2*H*)-yl)propanamide (3b)**

**IR-spectrum: *N*-Carbamimidoyl-2-(1-methyl-2,4-dioxo-1,4-dihydroquinazolin-3(2*H*)-yl)propanamide (3b)**

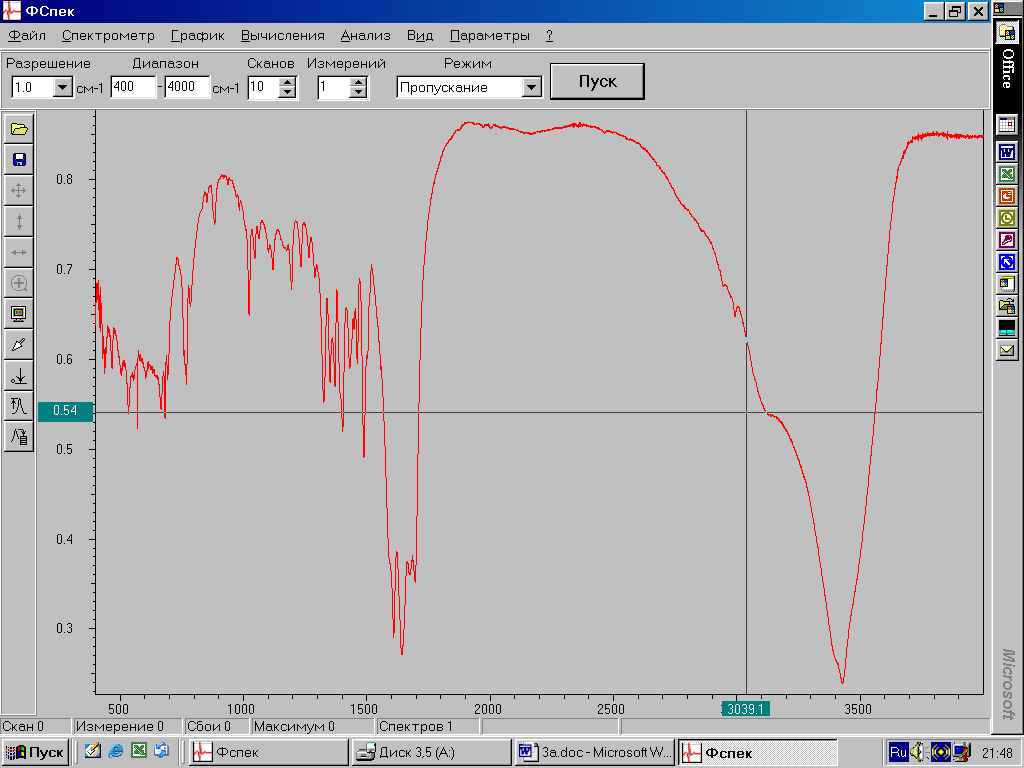


**^1^H NMR (DMSO-d_6_, 400 MHz): 2-(1-Benzyl-2,4-dioxo-1,4-dihydroquinazolin-3(2*H*)-yl)-*N*-carbamimidoylacetamide (3c)**

**^13^C NMR (DMSO-d_6_, 100 MHz): 2-(1-Benzyl-2,4-dioxo-1,4-dihydroquinazolin-3(2*H*)-yl)-*N*-carbamimidoylacetamide (3c)**

**IR-spectrum: 2-(1-Benzyl-2,4-dioxo-1,4-dihydroquinazolin-3(2*H*)-yl)-*N*-carbamimidoylacetamide (3c)**

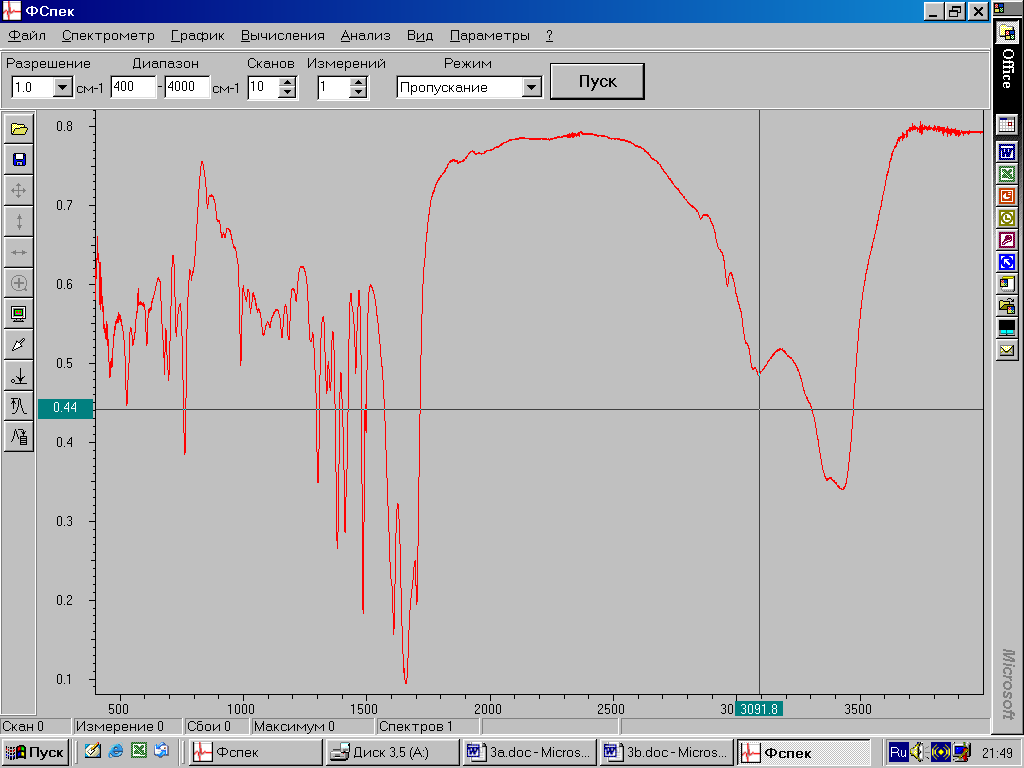


**^1^H NMR (DMSO-d_6_, 400 MHz): 2-(6-Bromo-1-methyl-2,4-dioxo-1,4-dihydroquinazolin-3(2*H*)-yl)-*N*-carbamimidoylacetamide (3d)**

**^13^C NMR (DMSO-d_6_, 100 MHz): 2-(6-Bromo-1-methyl-2,4-dioxo-1,4-dihydroquinazolin-3(2*H*)-yl)-*N*-carbamimidoylacetamide (3d)**

**IR-spectra: 2-(6-Bromo-1-methyl-2,4-dioxo-1,4-dihydroquinazolin-3(2*H*)-yl)-*N*-carbamimidoylacetamide (3d)**

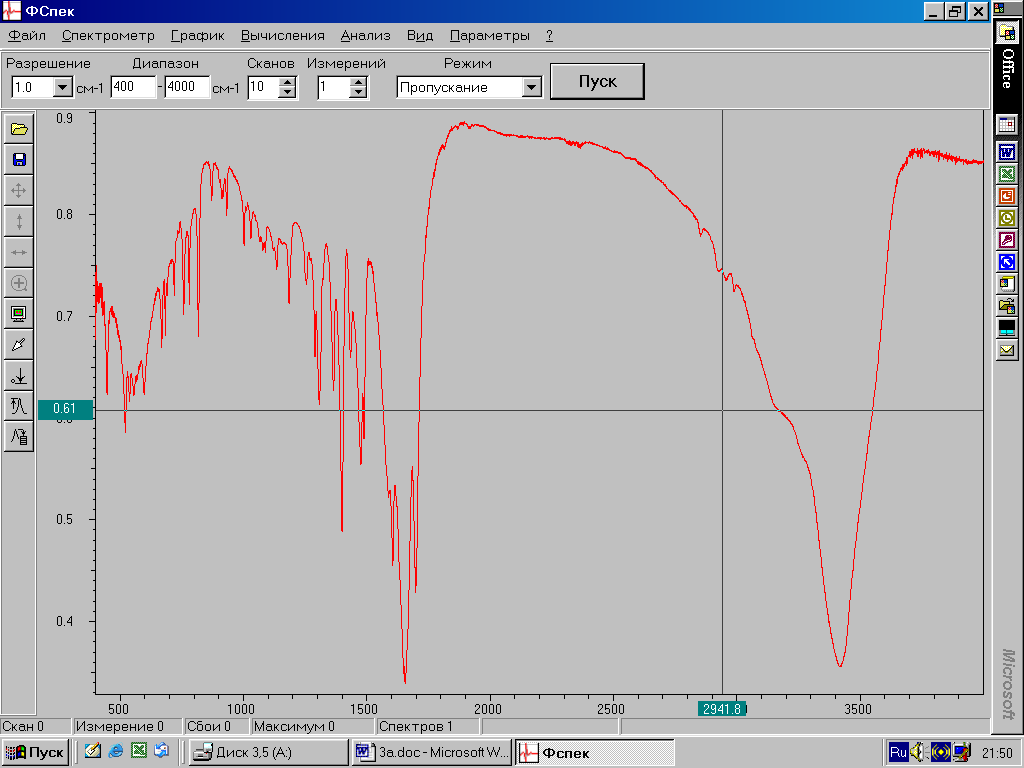


**^1^H NMR (DMSO-d_6_, 400 MHz): 3-[(5-Amino-4*H*-1,2,4-triazol-3-yl)methyl]-1-methylquinazoline-2,4(1*H*,3*H*)-dione (3e)**

**^13^C NMR (DMSO-d_6_, 100 MHz): 3-[(5-Amino-4*H*-1,2,4-triazol-3-yl)methyl]-1-methylquinazoline-2,4(1*H*,3*H*)-dione (3e)**

**IR-spectra: 3-[(5-Amino-4*H*-1,2,4-triazol-3-yl)methyl]-1-methylquinazoline-2,4(1*H*,3*H*)-dione (3e)**

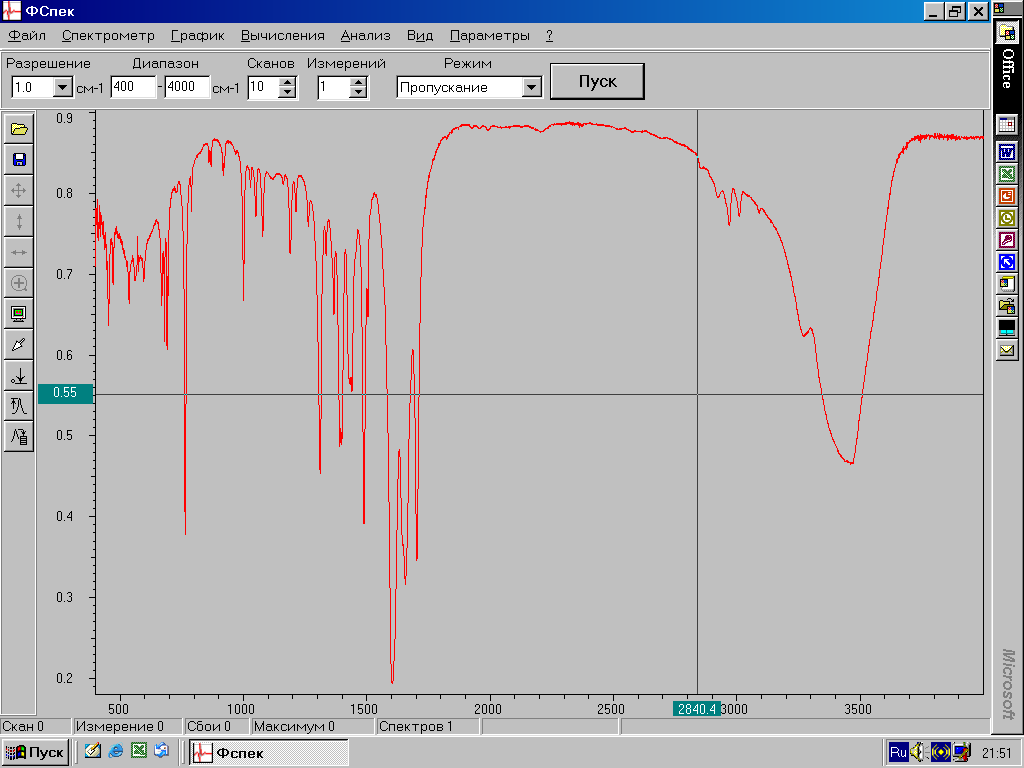


**^1^H NMR (DMSO-d_6_, 400 MHz): 3-[1-(5-Amino-4*H*-1,2,4-triazol-3-yl)ethyl]-1-methylquinazoline-2,4(1*H*,3*H*)-dione (3f)**

**^13^C NMR (DMSO-d_6_, 100 MHz): 3-[1-(5-Amino-4*H*-1,2,4-triazol-3-yl)ethyl]-1-methylquinazoline-2,4(1*H*,3*H*)-dione (3f)**

**IR-spectra: 3-[1-(5-Amino-4*H*-1,2,4-triazol-3-yl)ethyl]-1-methylquinazoline-2,4(1*H*,3*H*)-dione (3f)**

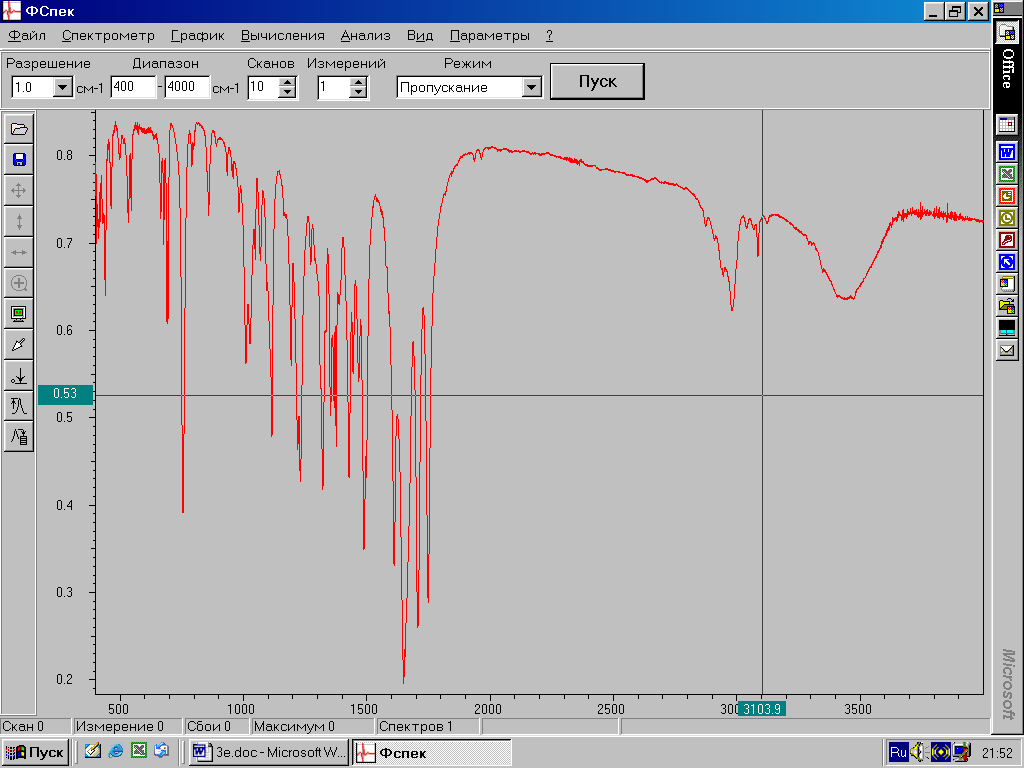


**^1^H NMR (DMSO-d_6_, 400 MHz): 3-[(5-Amino-4*H*-1,2,4-triazol-3-yl)methyl]-1-(prop-2-en-1-yl)quinazoline-2,4(1*H*,3*H*)-dione (3g)**

**^13^C NMR (DMSO-d_6_, 100 MHz): 3-[(5-Amino-4*H*-1,2,4-triazol-3-yl)methyl]-1-(prop-2-en-1-yl)quinazoline-2,4(1*H*,3*H*)-dione (3g)**

**IR-spectrum: 3-[(5-Amino-4*H*-1,2,4-triazol-3-yl)methyl]-1-(prop-2-en-1-yl)quinazoline-2,4(1*H*,3*H*)-dione (3g)**

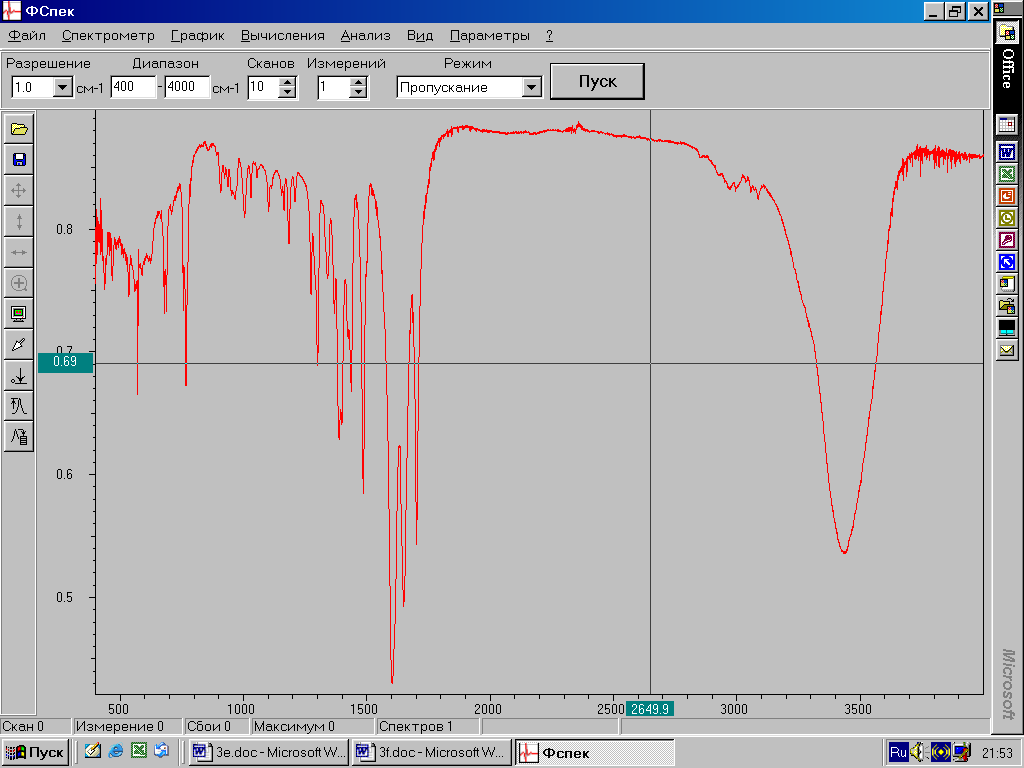


**^1^H NMR (DMSO-d_6_, 400 MHz): 3-[1-(5-Amino-4*H*-1,2,4-triazol-3-yl)ethyl]-1-(prop-2-en-1-yl)quinazoline-2,4(1*H*,3*H*)-dione (3h)**

**^13^C NMR (DMSO-d_6_, 100 MHz): 3-[1-(5-Amino-4*H*-1,2,4-triazol-3-yl)ethyl]-1-(prop-2-en-1-yl)quinazoline-2,4(1*H*,3*H*)-dione (3h)**

**IR-spectrum: 3-[1-(5-Amino-4*H*-1,2,4-triazol-3-yl)ethyl]-1-(prop-2-en-1-yl)quinazoline-2,4(1*H*,3*H*)-dione (3h)**

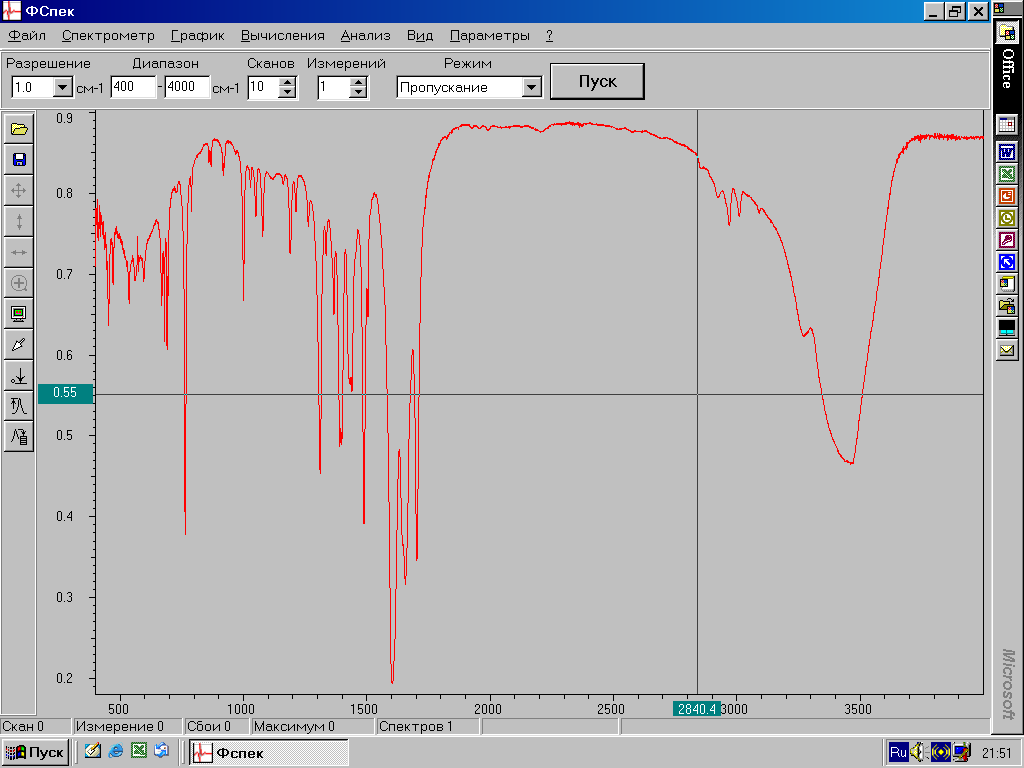


**^1^H NMR (DMSO-d_6_, 400 MHz): 3-[(5-Amino-4*H*-1,2,4-triazol-3-yl)methyl]-1-benzylquinazoline-2,4(1*H*,3*H*)-dione (3i)**

**^13^C NMR (DMSO-d_6_, 100 MHz): 3-[(5-Amino-4*H*-1,2,4-triazol-3-yl)methyl]-1-benzylquinazoline-2,4(1*H*,3*H*)-dione (3i)**

**IR-spectra: 3-[(5-Amino-4*H*-1,2,4-triazol-3-yl)methyl]-1-benzylquinazoline-2,4(1*H*,3*H*)-dione (3i)**

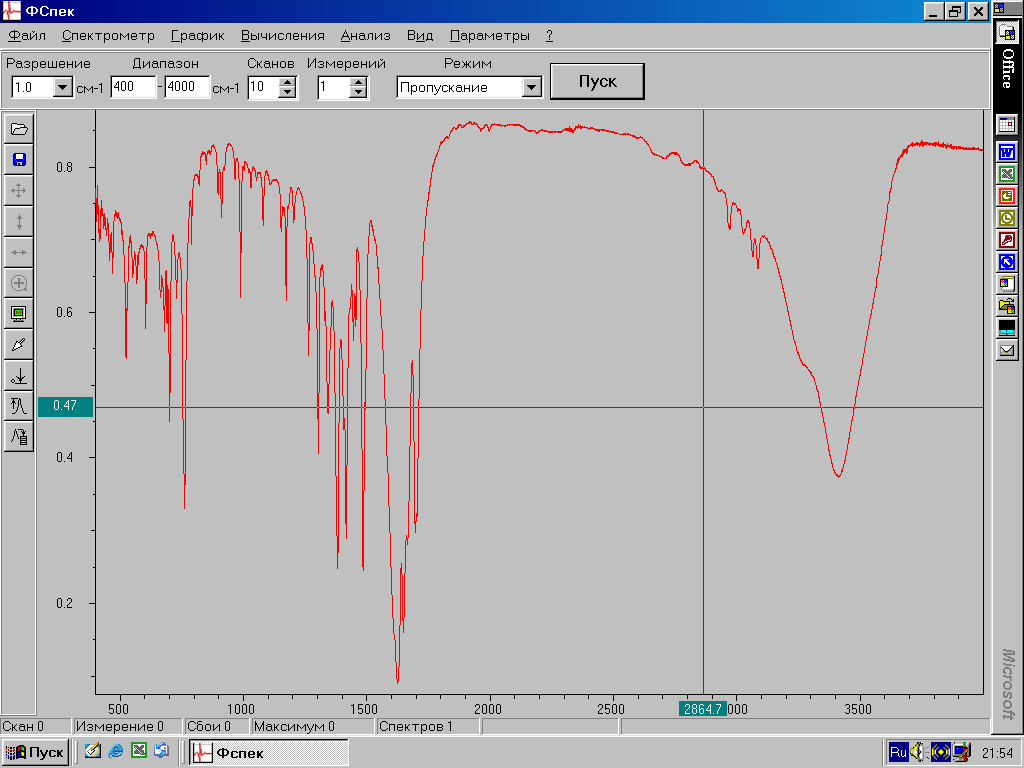


**^1^H NMR (DMSO-d_6_, 400 MHz): 3-[(5-Amino-4*H*-1,2,4-triazol-3-yl)methyl]quinazolin-4(3*H*)-one (6a)**

**^13^C NMR (DMSO-d_6_, 100 MHz): 3-[(5-Amino-4*H*-1,2,4-triazol-3-yl)methyl]quinazolin-4(3*H*)-one (6a)**

**IR-spectrum: 3-[(5-Amino-4*H*-1,2,4-triazol-3-yl)methyl]quinazolin-4(3*H*)-one (6a)**

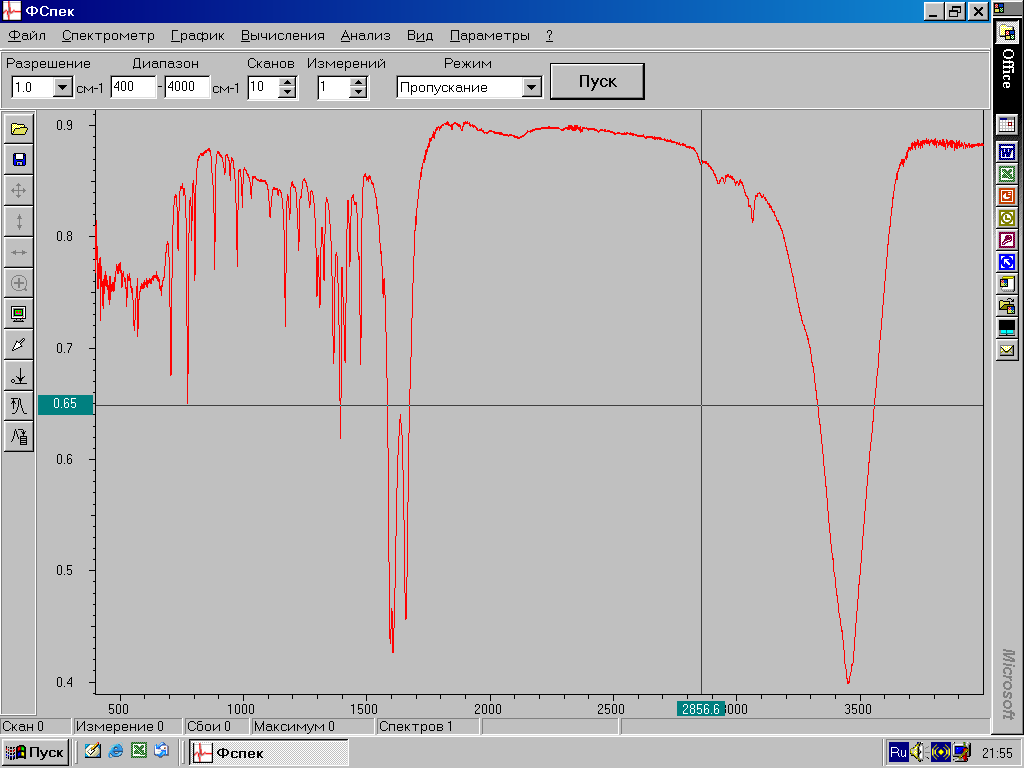


**^1^H NMR (DMSO-d_6_, 400 MHz): 3-[(5-Amino-1*H*-1,2,4-triazol-3-yl)methyl]-6-bromoquinazolin-4(3*H*)-one (6b)**

**^13^C NMR (DMSO-d_6_, 100 MHz): 3-[(5-Amino-1*H*-1,2,4-triazol-3-yl)methyl]-6-bromoquinazolin-4(3*H*)-one (6b)**

**IR-spectra: 3-[(5-Amino-1*H*-1,2,4-triazol-3-yl)methyl]-6-bromoquinazolin-4(3*H*)-one (6b)**

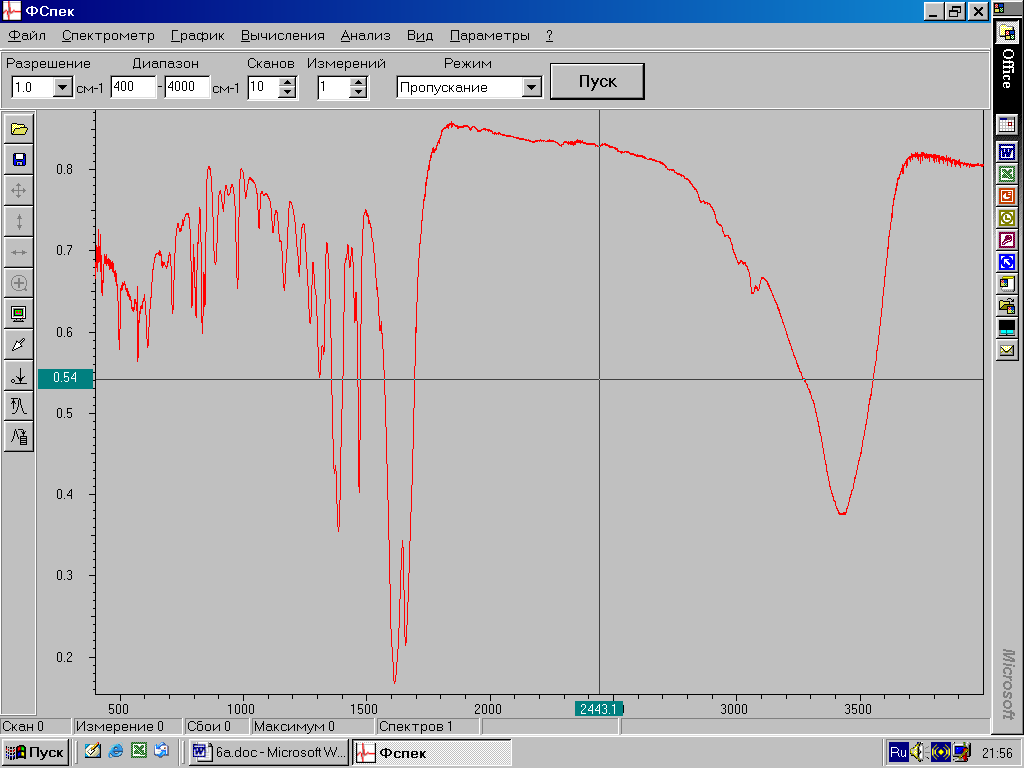

Supplement: Supplementary file 1 — Supplementary Information. [file 41598_2021_3722_MOESM1_ESM.docx]
